# Wnt signaling is boosted during intestinal regeneration by a CD44-positive feedback loop

**DOI:** 10.1038/s41419-022-04607-0

**Published:** 2022-02-21

**Authors:** Romina J. Walter, Steffen J. Sonnentag, Leonel Munoz-Sagredo, Melanie Merkel, Ludovic Richert, Felix Bunert, Yvonne M. Heneka, Thomas Loustau, Michael Hodder, Rachel A. Ridgway, Owen J. Sansom, Yves Mely, Ulrich Rothbauer, Mark Schmitt, Véronique Orian-Rousseau

**Affiliations:** 1 Institute of Biological and Chemical Systems - Functional Molecular Systems (IBCS-FMS), Karlsruhe Institute of Technology (KIT), Hermann-von-Helmholtz-Platz 1, 76344 Eggenstein-Leopoldshafen, Germany; 2 Faculty of Medicine, Universidad de Valparaiso, Angamos 655, 2540064 Vina del Mar, Chile; 3 UMR 7021 CNRS, Laboratoire de Bioimagerie et Pathologies, Faculté de Pharmacie, 74 route du Rhin, 67401 Illkirch, France; 4 Cancer Research UK Beatson Institute, Garscube Estate, Switchback Road, Glasgow, G61 1BD UK; 5 Institute of Cancer Sciences, University of Glasgow, Garscube Estate, Switchback Road, Glasgow, G61 1QH UK; 6 NMI Natural and Medical Sciences Institute at the University of Tuebingen, Markwiesenstrasse 55, 72770 Reutlingen, Germany; 7 Pharmaceutical Biotechnology, Eberhard Karls University Tuebingen, Auf der Morgenstelle 8, 72076 Tübingen, Germany; 8 Institute of Pharmacology, University of Marburg, Karl-von-Frisch-Strasse 2, 35043 Marburg, Germany

**Keywords:** Intestinal stem cells, Self-renewal

## Abstract

Enhancement of Wnt signaling is fundamental for stem cell function during intestinal regeneration. Molecular modules control Wnt activity by regulating signal transduction. CD44 is such a positive regulator and a Wnt target gene. While highly expressed in intestinal crypts and used as a stem cell marker, its role during intestinal homeostasis and regeneration remains unknown. Here we propose a CD44 positive-feedback loop that boosts Wnt signal transduction, thus impacting intestinal regeneration. Excision of *Cd44* in *Cd44*^*fl/fl*^*;VillinCreER*^*T2*^ mice reduced Wnt target gene expression in intestinal crypts and affected stem cell functionality in organoids. Although the integrity of the intestinal epithelium was conserved in mice lacking CD44, they were hypersensitive to dextran sulfate sodium, and showed more severe inflammation and delayed regeneration. We localized the molecular function of CD44 at the Wnt signalosome, and identified novel DVL/CD44 and AXIN/CD44 complexes. CD44 thus promotes optimal Wnt signaling during intestinal regeneration.

## Introduction

The fast self-renewal of the intestinal epithelium is carried out by intestinal stem cells (ISCs) that lie at the bottom of the intestinal crypts and express leucine-rich repeat G-protein coupled receptor 5 (Lgr5). These cells give rise to transit-amplifying (TAs) cells that differentiate into enterocytes or secretory lineages like enteroendocrine cells (EECs), goblet cells (GCs), and Paneth cells (PCs) [1].

The Wnt signaling pathway is fundamental in intestinal homeostasis and regeneration after damage [1]. In the ISC niche of the small intestine, Wnt ligands are produced by PCs—which form part of the niche and need high Wnt signaling levels for their own maturation [2]—and by cells of the stroma surrounding the crypt bottom [3]. The activity of Wnt is the highest in the intestinal stem cell niche at the bottom of the crypts, where it acts as the major regulator of ISC self-renewal and differentiation, and decreases in a gradient along the crypt–villus axis [1, 4].

However, Wnt levels are not static and require increments for regenerative processes after intestinal injury [5]. Increases in ligand secretion need to be coupled to efficient signal transduction dependent on receptor availability and optimal function, regulated by different molecular modules. An example is the R-Spondin/Lgr4/5 axis, which stabilizes the Wnt receptor Frizzled (FZD) at the cell membrane [6–8]. Upon Wnt ligand binding to FZD and LRP6, a multiprotein signaling complex—the Wnt signalosome—is assembled at the membrane, immobilizing the β-catenin destruction complex. β-catenin translocates to the nucleus activating the TCF/LEF-mediated Wnt transcriptional program [9].

Signalosome components that are at the same time Wnt target genes can act as feedback loops amplifying or inhibiting signal transduction. CD44 is highly expressed in the intestinal crypt cells and is a Wnt target gene [10]. We previously showed that it acts as a positive regulator for canonical Wnt signaling at the receptor level [11]. In the present study, we explored the impact of CD44 on the regulation of Wnt signaling during homeostasis of the intestinal epithelium and regeneration upon injury.

*Cd44* knockout from the intestinal epithelium in *Cd44*^*fl/fl*^*;VillinCreER*^*T2*^ mice reduced nuclear β-catenin in the intestinal crypts, decreasing the expression of Wnt pathway target genes, and resulted in a decreased number of PCs. Stem cell functionality was impaired in *Cd44*^*-/-*^ intestinal organoids. Upon dextran sulfate sodium (DSS)-induced colitis, these mice had increased inflammation and delayed regeneration. At the molecular level, proximity ligation assays (PLA) and co-immunoprecipitations with LRP6, DVL, and AXIN, and sucrose gradients, demonstrate that CD44 forms part of the Wnt-induced signalosome. FRET-FLIM experiments revealed direct contact between CD44 and LRP6 at discrete locations of the cell membrane. Taken together, these results suggest a positive feedback function of CD44 that boosts the Wnt signaling pathway during high Wnt signaling demand, as required for intestinal regeneration.

## Results

### Deletion of *Cd44* in the intestinal epithelium led to a reduction in Wnt signaling in the crypts

To evaluate the function of CD44 in intestinal homeostasis and regeneration, we used *Cd44*^*fl/fl*^*;VillinCreER*^*T2*^ mice in which *Cd44* is specifically removed from the intestinal epithelium. These mice, when treated with tamoxifen, are designated *Cd44*^*Δie*^ throughout the paper. *Cd44*^*fl/fl*^ mice, not carrying the *CreER*^*T2*^ transgene, were used as control mice (designated *Cd44*^*+/+*^). Upon deletion of *Cd44* in the intestinal epithelium, the gross morphology and crypt/villus tissue architecture of the small intestine (SI) and colon observed in hematoxylin and eosin staining was not altered (Supplementary Fig. 1A). Immunofluorescence staining using an antibody against CD44 confirmed its expression in the crypts of the small and large intestine and in the *lamina propria* of *Cd44*^*+/+*^ mice (Supplementary Fig. 1Ba/a’, Be/e’) [10, 12]. The same observations could be made in the *Cd44*^*fl/fl*^*;VillinCreER*^*T2*^ mice not treated with tamoxifen used as an additional control (Supplementary Fig. 1Bc/c’, Bg/g’). In the *Cd44*^*Δie*^ mice, CD44 was exclusively removed from the crypts, while the expression in cells of the *lamina propria* (arrowheads) was maintained (Supplementary Fig. 1Bb/b’, Bf/f’). The absence of *Cd44* was observed up to 38 days post-tamoxifen treatment (Supplementary Fig. 1Bd/d’, Bh/h’).

**Fig. 1:**
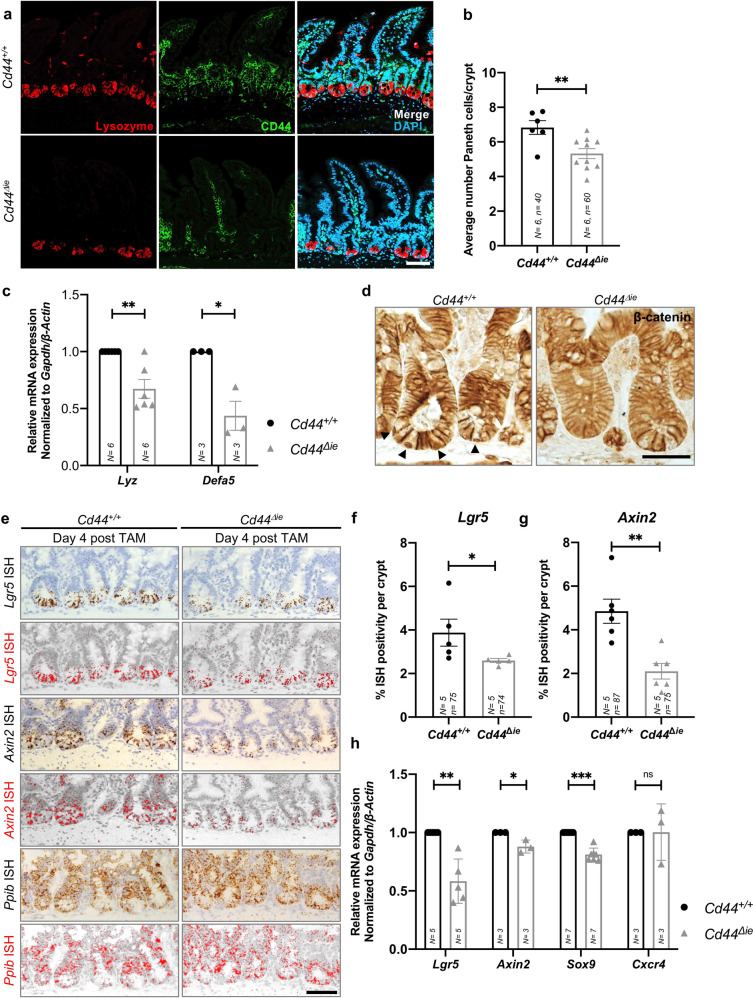
Deletion of epithelial *Cd44* reduces Wnt signaling in murine intestinal crypts. **a** Confocal image of IF staining on sections of the small intestine (SI) of *Cd44*^*Δie*^ and *Cd44*^*+/+*^ mice using anti-lysozyme antibodies. Scale bar: 50 µm. **b** Quantification of lysozyme-stained PCs in the crypts of *Cd44*^*Δie*^ and *Cd44*^*+/+*^ mice. Error bars, ±SE. **c** qPCR of *lysozyme* and *α-defensin5 (Lyz* and *Defa5)* expression in intestinal crypts from *Cd44*^*Δie*^ and *Cd44*^*+/+*^ mice. *Gapdh* and *β-Actin* were used as reference genes. Error bars, ±SE. **d** Nuclear β-catenin (arrowheads) IHC staining. Scale bar: 25 μm. **e** In situ hybridization (ISH) (brown, red) of *Lgr5* and *Axin2* expression in the SI. Positive control: *Ppib*. Error bars, ± SE. Scale bar: 50 µm. **f**, **g** Quantification of ISH. Error bars, ±SE. **h** qPCR of Wnt target genes and stem cell marker expression in SI crypts isolated from *Cd44*^*Δie*^ and *Cd44*^*+/+*^ mice. Non-Wnt-regulated control: *Cxcr4*. Error bars, ±SE. Student’s *t* test; *p < 0.05, **p < 0.01, ***p < 0.001, ns not significant. *N* = number of mice, *n* = number of crypts.

Since Wnt controls stemness during the renewal of the intestinal epithelium, we evaluated the differentiation capacity of ISCs by examination of secretory lineages—GCs, EECs, and PCs—after deletion of *Cd44* in the SI. We detected no significant difference in the number of GCs and EECs in the SI of *Cd44*^*Δie*^ compared to *Cd44*^*+/+*^ mice (Supplementary Fig. 2A–D). PCs were stained with antibodies against lysozyme. In contrast to the other cell types, lysozyme-positive cells were reduced in number following *Cd44* deletion (Fig. 1a, b). *Lyz* and *Defa5* mRNA levels were also significantly decreased in the crypts (Fig. 1c) supporting the observation that the loss of *Cd44* in the intestinal epithelium led to a reduction in the number of PCs. Of note, this reduction was neither due to apoptosis in the crypt nor due to reduced proliferation (Supplementary Fig. 2E–H).

Wnt/β-catenin signaling induces differentiation of PCs, thus supporting their maturation and positioning at the crypt base [13]. We therefore examined whether the decreased number of PCs in the absence of CD44 might be related to its role in the Wnt-signaling pathway [11]. We assessed the nuclear localization of β-catenin in crypt cells by immunohistochemistry and observed a clear reduction of cells with nuclear β-catenin (Fig. 1d arrowheads) in the SI and reduced levels of β-catenin in the colon (Supplementary Fig. 2I, J) of *Cd44*^*Δie*^ mice. To determine the spatial expression level of Wnt target genes in the intestinal epithelium, we performed in situ hybridization (ISH) using probes of *Axin2* and *Lgr5* on paraffin sections of the SI of *Cd44*^*Δie*^ and *Cd44*^*+/+*^ mice, sacrificed 4 days post TAM induction. As seen in Fig. 1e–g, the ISH staining decreased significantly in *Cd44*^*Δie*^ mice. *Ppib* (Peptidyl-prolyl cis-trans isomerase B) was used as a negative control [14]. Supporting these results, mRNA levels of the Wnt target genes *Lgr5*, *Axin2*, and *Sox9* in intestinal epithelial crypts of *Cd44*^*Δie*^ were decreased as well. In comparison, the level of *Cxcr4*, which is not a Wnt target gene, remained unchanged (Fig. 1h). These data indicate that *Cd44* deletion reduces Wnt signaling in the murine intestinal epithelium. Interestingly, MAPK signaling was apparently not increased in the crypt bottom (Supplementary Fig. 2K) as described in the case of reduced Wnt signaling by orcupine inhibition [15].

**Fig. 2:**
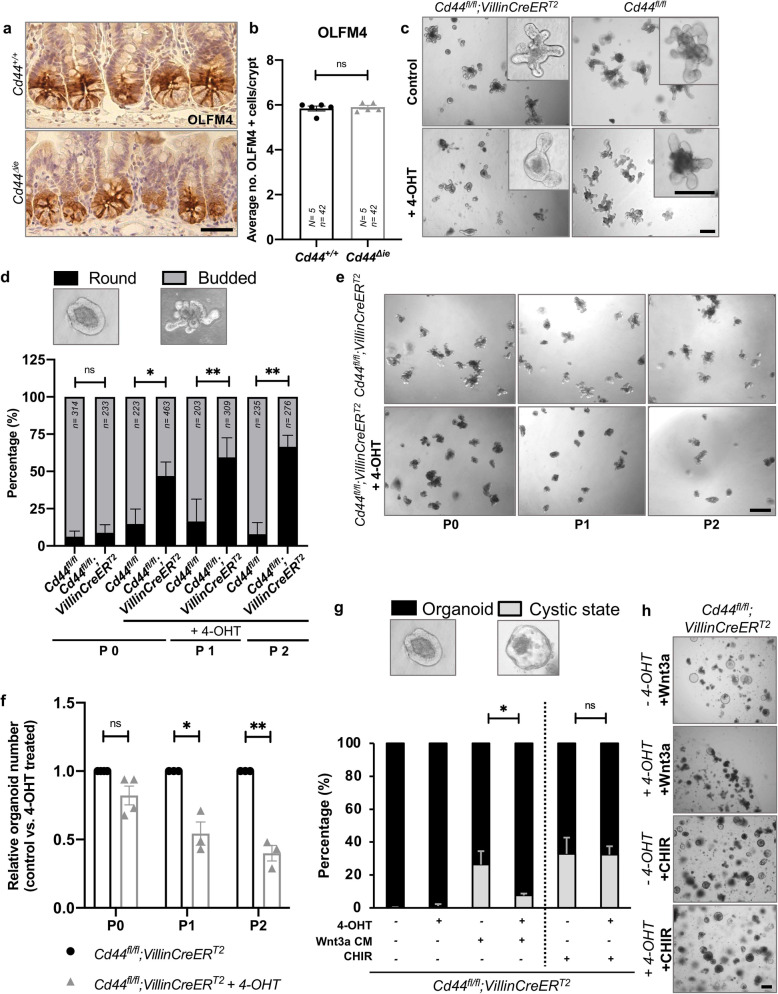
Conserved numbers but impaired functionality of stem cells in *Cd44*^*Δie*^ mice. **a** IHC staining of OLFM4. Scale bar: 50 μm. **b** Average number of OLFM4-positive stem cells per crypt. Error bars, ±SE. *N* = number of mice, *n* = number of crypts. **c** SI organoids observed 5 days after *Cd44* deletion and controls. Scale bar: 200 µm. **d** Budded vs. round organoid numbers after deletion of *Cd44* and passaging (P) every 5 days. *n* = number of organoids. Error bars, ±SE. **e** Representative images of **f**. Scale bar: 200 µm. **f** Relative organoid number along passages in *Cd44*^*fl/fl*^*;VillinCreER*^*T2*^ organoid cultured with or without 4-OHT treatment. *N* = 3 mice. Scale bar: 500 µm. **g** Organoid vs. cystic organoid numbers after deletion of *Cd44* and treatment with Wnt3a CM, control CM or CHIR. *N* = 3 experiments. Error bars, ±SE. **h** Representative images of **g** CHIR and Wnt3a conditions. Scale bar: 200 µm. Student’s *t* test; *p < 0.05, **p < 0.01, ns = not significant. 4-OHT 4-hydroxytamoxifen. The dotted line in **g** indicates that the CHIR and Wnt3a conditions are two independent but related experiments.

Since the deletion of *Cd44* led to a reduction of the Wnt pathway activation, we assessed its impact on the ISC pool. Surprisingly, *Cd44* deletion did not affect the quantity of OLFM4 positive crypt base columnar (CBC: brown thin cells) cells in *Cd44*^*Δie*^ mice in SI (Fig. 2a, b). However, the impaired expression of the Wnt and stem cell markers *Lgr5*, *Axin2*, and *Sox9* in CBC cells point towards an impaired functionality of CBC stem cells.

### *Cd44* deletion in small intestinal organoids impairs stem cell functionality

To address ISC functionality we performed small intestinal organoid assays. ISCs form organoid structures ex vivo in a Wnt-dependent manner [16] and give rise to all cell types of the intestinal epithelium. During their differentiation, these organoids form buddings that mimic the intestinal crypt and contain Lgr5^+^ stem cells. Of note, the development and budding of intestinal organoids are dependent on the activity of the Wnt pathway as well as on a functional interplay between Lgr5^+^ stem cells and PCs [17, 18]. We isolated SI crypts of *Cd44*^*fl/fl*^*;VillinCreER*^*T2*^ and *Cd44*^*fl/fl*^ mice (not treated with TAM) and cultured them in the presence of 4-hydroxytamoxifen (4-OHT) in order to observe the effect of the knockout of *Cd44* on organoid formation and differentiation.

As shown in Fig. 2c, deletion of *Cd44* (Supplementary Fig. 3A) impaired SI organoid budding. Although we observed some buddings in the *Cd44*-deleted organoids, they were reduced in number. In addition, passaging of organoids deficient of *Cd44* resulted in more frequent complete inhibition of organoid budding (round organoids) (Fig. 2d) as well as reduced numbers of organoids (Fig. 2e, f). This indicates decreased stem cell potency and self-renewal upon *Cd44* deletion, most likely due to reduced Wnt signaling. Similar effects could be observed in colonic organoids (Supplementary Fig. 3B, C). Lack of *Cd44* resulted in reduced survival of colon organoids after passaging suggesting that the effect of the removal of *Cd44* on ISCs is not due to the decrease in PC number since these cells are not found in the colon.

Hyperactivation of the Wnt pathway in small intestinal organoids leads to a drastic morphological change in which organoids transform from a budded structure to a cystic state, reflecting a less differentiated state compared to budded organoids [19]. Notably, organoid treatment with the GSK-3 inhibitor CHIR99021 (CHIR) and potent activator of the Wnt pathway downstream of the membrane receptors i.d. downstream of the regulatory function of CD44 in the Wnt-cascade, resulted in an equal number of organoids in the cystic state in CD44-deficient and CD44-proficient organoids (Fig. 2g, h). In contrast, activation at the receptor level using the Wnt3a ligand resulted in a reduced number of cystic stage organoids upon removal of *Cd44* (Fig. 2g, h). Altogether, these results indicate that the Wnt pathway activation in intestinal organoids is impaired upon *Cd44* deletion when the activation occurs at the receptor level, whereas activation downstream remains unaffected.

### *Cd44* deletion in the intestinal epithelium increases the severity of DSS-induced colitis

Even though the residual Wnt signaling after *Cd44* deletion seems to be sufficient to maintain the ISC number and intestinal homeostasis in vivo, tissue regeneration after damage, a process that requires higher Wnt activity to increase stem cell function, may not be sufficiently supported [20, 21].

**Fig. 3:**
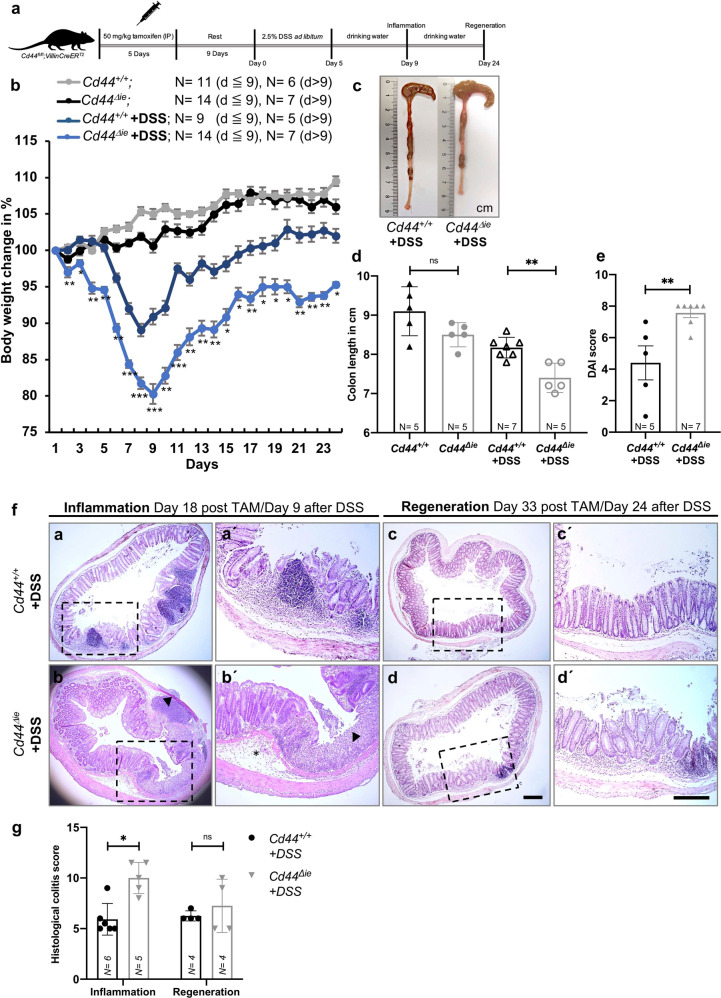
DSS-treated *Cd44*^*Δie*^ mice had increased intestinal inflammation and delayed regeneration. **a** Experimental design. **b** Daily body weight change (% of initial body weight) of *Cd44*^*Δie*^ and *Cd44*^*+/+*^ mice subjected to 2.5% DSS or normal drinking water. The mean weight difference of the two DSS treated groups (*Cd44*^*Δie*^ vs. *Cd44*^*+/+*^ mice) was compared for every timepoint separately. Error bars, ±SE. **c** Representative images of colons from DSS-treated *Cd44*^*Δie*^ and *Cd44*^*+/+*^ mice. **d** Colon lengths from *Cd44*^*Δie*^ and *Cd44*^*+/+*^ mice +/− DSS. Error bars, ±SE. **e** Disease activity index (DAI) of the mice described in **b**. Error bars, ±SE. **f** H&E staining of colon sections of the mice described in **b**. Scale bar: 200 µm. Asterisk: submucosa. Arrowheads: mucosa (a’–d’ is an enlargement of a–d). **g** Histological colitis score (HCS) of the mice described in **b**. Error bars, ±SE. Student’s *t* test; *p < 0.05, **p < 0.01, ***p < 0.001, ns = not significant, except Fig. 3e, g: Mann–Whitney *U*-test. IP intraperitoneal, TAM tamoxifen, *N* = number of mice.

To study the impact of CD44 in intestinal regeneration, *Cd44*^*fl/fl*^*;VillinCreER*^*T2*^ and *Cd44*^*fl/fl*^ mice, were injected with TAM and thereafter given a 2.5% DSS solution or normal drinking water as a control (Fig. 3a). Nine days after the start of DSS administration, the mice were sacrificed to analyze the severity of inflammation. Regeneration of the intestinal epithelium was analyzed at day 24, a time point at which DSS-treated *Cd44*^*+/+*^ mice had already regained their initial weight (Fig. 3a, b). As shown in Fig. 3b, *Cd44*^*Δie*^ mice treated with DSS lost more weight than the control mice, suggesting more severe intestinal damage. Therefore, intestinal regeneration took longer in *Cd44*^*Δie*^ mice than in *Cd44*^*+/+*^ mice, and was not complete at the last analysis time point (24 days after DSS initiation). In contrast, the *Cd44*^*+/+*^ mice had regained their initial weight at day 16 after DSS initiation (Fig. 3b).

The more severe weight loss upon *Cd44* deletion in DSS-treated mice was associated with a shortening of the colon indicating an increased inflammatory response (Fig. 3c, d). The disease activity index (DAI), which includes the percentage of body weight loss, stool consistency, and rectal bleeding [22], was higher in *Cd44*^*Δie*^ mice than in *Cd44*^*+/+*^ mice treated with DSS (Fig. 3e). Nine days after the start of DSS treatment, *Cd44*^*Δie*^ mice had a higher histological colitis score (HCS) [23]. After epithelial regeneration, the overall architecture was largely restored, with areas showing remaining tissue inflammation in mice lacking *Cd44* (Fig. 3f, g).

**Fig. 4:**
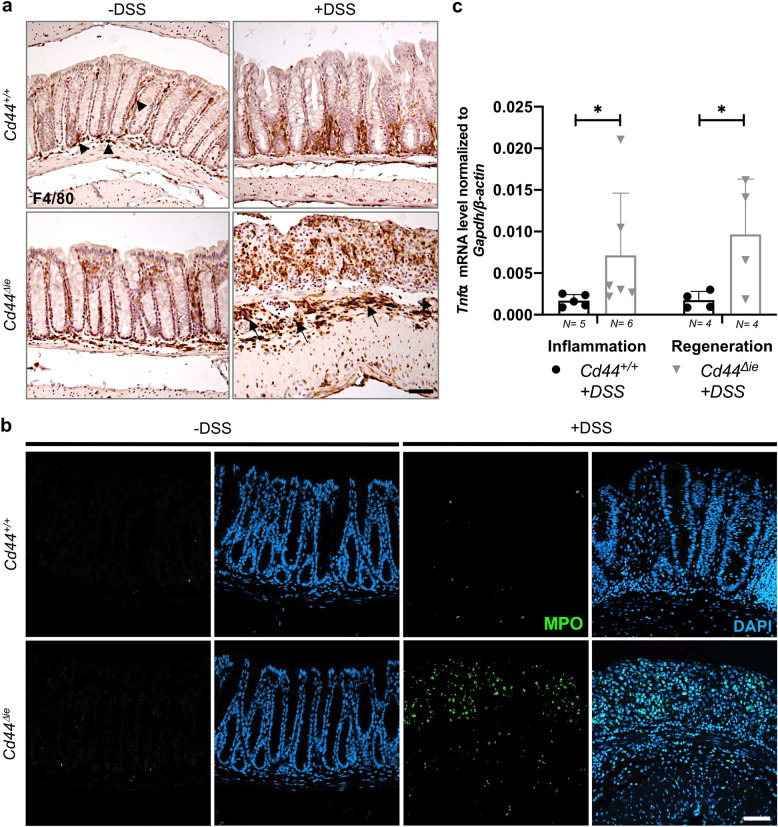
*Cd44*^*Δie*^ mice exhibit higher innate immune cell infiltration upon DSS-induced colitis. **a** IHC staining of F4/80 (macrophages) on colon sections. Arrowheads: mucosa, Arrows: submucosa. Scale bar: 50 µm. **b** IF staining of myeloperoxidase (MPO) (neutrophils) on colon sections. Scale bar: 50 µm. **c** qPCR of inflammation marker *Tnfα* expression in colon samples from *Cd44*^*Δie*^ and *Cd44*^*+/+*^ mice. Reference genes: *Gapdh* and *β-Actin*. Error bars, ±SE. Inflammation: Mann–Whitney *U*-test. Regeneration: Student’s *t* test with unequal variances; *p < 0.05. *N* = number of mice.

DSS-mediated colitis is characterized by the infiltration of immune cells with the consequent secretion of mediators like chemokines and cytokines [24]. After DSS, macrophage and neutrophil infiltration detected in *Cd44*^*Δie*^ mice was drastically higher compared to controls, especially in the submucosa (arrows) (Fig. 4a, b). Consequently, *Cd44*^*Δie*^ mice had significantly higher mRNA levels of the inflammation marker *Tnfα* not only at the time point of the most severe inflammation but also during regeneration (Fig. 4c).

**Fig. 5:**
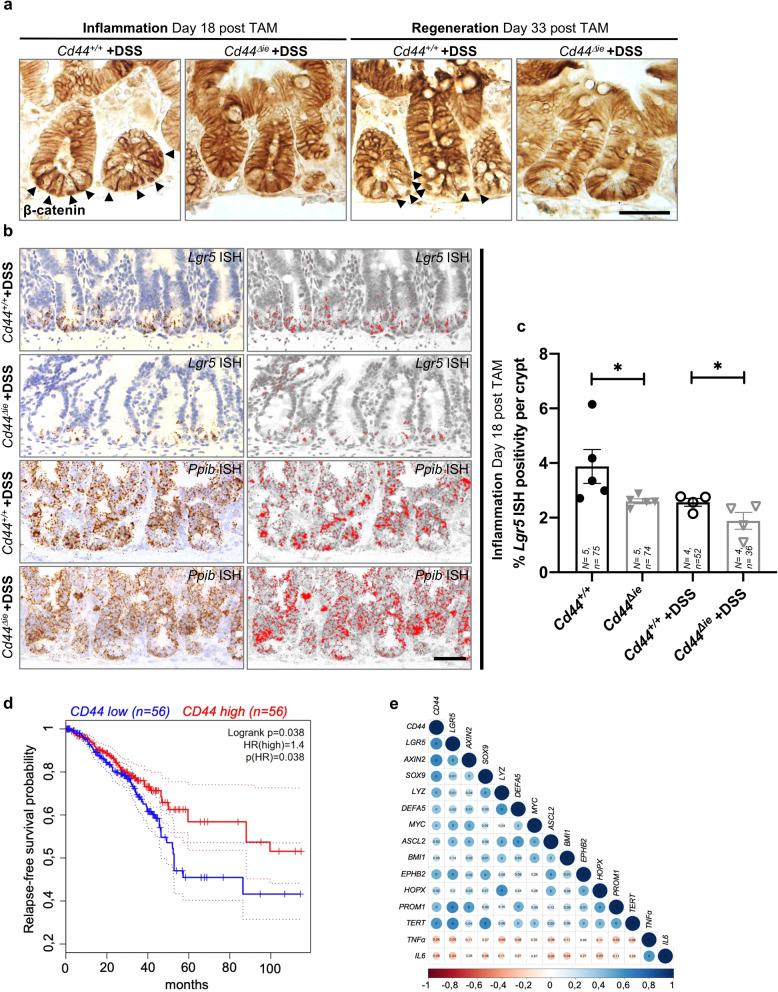
Mice treated with DSS exhibit reduced Wnt signaling in the small intestine—CD44 influences IBD in humans. **a** IHC staining using antibodies against β-catenin. Arrowheads: nuclear β-catenin (*N* = 3 mice). Scale bar: 50 μm. **b** In situ hybridization (ISH) (brown, red) of *Lgr5* Wnt target gene expression in the SI of *Cd44*^*Δie*^ and *Cd44*^*+/+*^ mice treated with DSS. Positive control: *Ppib*. Scale bar: 50 µm. **c** Quantification of ISH staining. Error bars, ±SE. Student’s *t* test, (*p < 0.05). TAM tamoxifen, *N* = number of mice, *n* = number of crypts. **d** Kaplan–Meier analysis of Crohn’s disease patients (GSE137344) relapse-free survival and expression of CD44 above or below the median. Hazard ratio (HR) = 1.4; *p* = 0.038; *n* = 112; dotted lines = SD. **e** Corrplot package analysis for visualization of the correlation matrix between CD44 and the indicated genes in Crohn’s disease patients (GSE171244). The values in the circles are the adjusted *p* values and the color intensities correspond to Spearman correlation values. Adjusted *p* values < 0.01 are annotated as 0. Sizes of the circle are proportional to the *p* value.

The severity of the inflammatory response and the time needed for recovery after DSS is directly related to the epithelial permeability to luminal bacteria, which is dependent on the regenerative capability of the mucosal lining [25, 26]. This process is driven by ISC function, which is dependent on high levels of Wnt signaling [27, 28]. We previously showed that CD44 acts as a Wnt signaling activity regulator [11]. Therefore, we hypothesized that the increased inflammatory effect of DSS, after *Cd44* deletion in the intestine, is due to a delayed regeneration capability, which in turn results from reduced Wnt signaling activity in the ISCs. After DSS treatment, we also found a reduction in the accumulation of nuclear β-catenin in crypt cells of the SI in *Cd44*^*Δie*^ mice compared to *Cd44*^*+/+*^ mice (Fig. 5a). Moreover, ISH revealed that although *Lgr5* expression in *Cd44*^*+/+*^ was reduced compared to non-DSS treated mice, this reduction was significantly more severe in the small intestinal crypts of *Cd44*^*Δie*^ mice after DSS treatment (Fig. 5b, c). This indicates a defect of stem cell regeneration after DSS-induced inflammation. In summary, the in vivo knockout of *Cd44* shows a phenotype upon DSS-induced damage, characterized by increased severity of the inflammatory response and delayed recovery, most likely associated with decreased Wnt signaling activity in *Cd44*-deficient mice.

### CD44 levels influence the progression of intestinal bowel disease in humans

By investigating publicly available gene expression data (GEO database, GSE137344 [29]), we assessed the expression levels of *CD44* in Crohn’s disease (CD) patients. Low *CD44* expression (below the median) correlated significantly with a shorter time of relapse-free survival whereas high *CD44* expression correlated with sustained remission in this patient cohort (Fig. 5d). Since CD is associated with a compromised epithelial barrier function, these data support the role of CD44 in sustaining the mucosal integrity in a condition susceptible to chronic inflammation.

Coherently, the study of RNAseq data from 10 CD patients (GEO database, GSE171244 [30]) shows a positive correlation between the expression levels of *CD44* and Wnt target genes, PCs, and stem cell markers (Fig. 5e). On the other hand, this expression profile negatively correlated with pro-inflammatory markers like *TNFα* and *IL-6*, suggesting that prolonged relapse-free survival of CD patients is associated with high Wnt signaling during repair and maintenance of epithelial integrity upon damage.

### CD44 forms part of the Wnt signalosome and directly interacts with LRP6

**Fig. 6:**
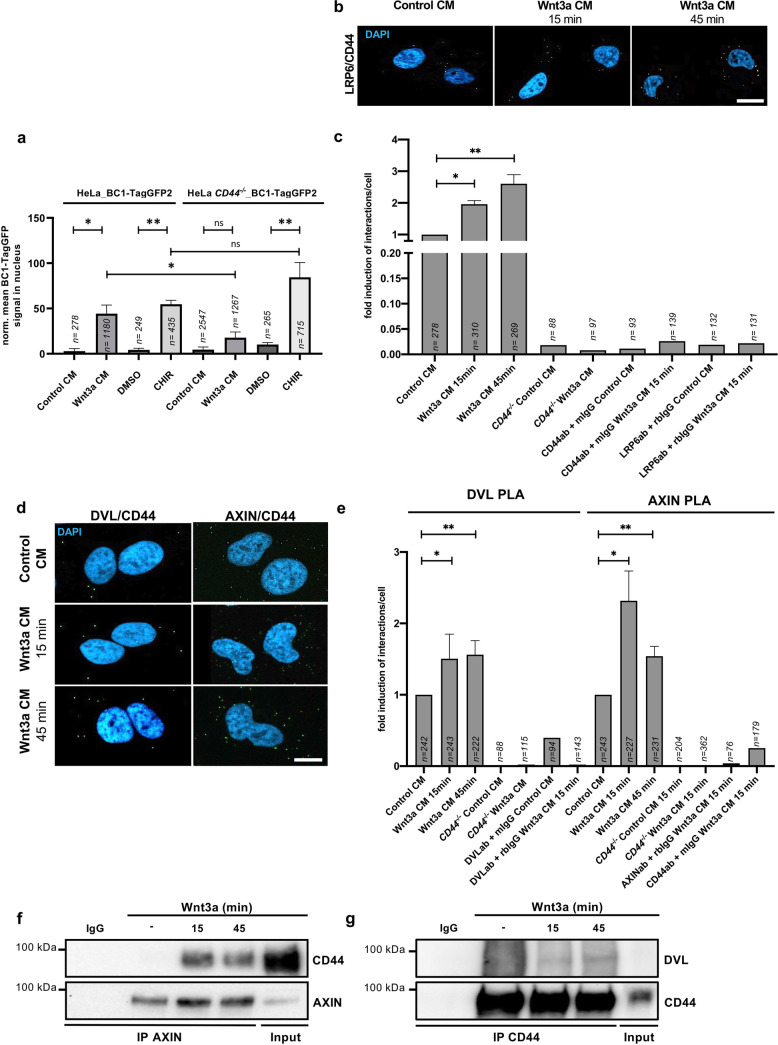
CD44 is in complex with LRP6, AXIN, and DVL after Wnt induction. **a** Mean fluorescence of chromobodies (CB) against active β-catenin in the nucleus of DMSO, CHIR, control CM, or Wnt3a CM-treated HeLa_BC1-TagGFP2 or *CD44*^*−/−*^_HeLa_BC1-TagGFP2 cells 24 h after induction. Control CM vs. Wnt3a CM: Mann–Whitney *U*-test. DMSO vs. CHIR: Student’s *t* test. Error bars, ±SE. **b**, **c** CD44/LRP6, CD44/DVL, and CD44/AXIN **d**, **e** in situ PLA complexes (dots) in HeLa cells treated with Wnt3a CM for 15 and 45 min, compared to control CM-treated cells. Cells were incubated with primary antibodies against CD44 and LRP6, DVL, or AXIN. Error bars, ±SE. Scale bar: **b** 20 μm, **d** 10 μm. **c**, **e** Fold induction of average interactions per cell, compared to control CM treatment. Error bars, ±SE. Student’s *t* test. **f**, **g** Co-immunoprecipitation of AXIN/CD44 (**f**) and CD44/DVL (**g**) on lysates of HeLa cells induced with Wnt3a CM for the indicated time points. Student’s *t* test/Mann–Whitney *U*-test: *p < 0.05, **p < 0.01, ns not significant. *n* = number of analyzed cells.

The above data suggest that CD44 has a role in ISC functionality. We had previously shown that CD44 knockdown in HEK293T cells reduces Wnt signaling activity as seen by TOPFlash assays [11]. Similarly, CRISPR/Cas9 knockout of *CD44* in NCI-H1703 cells (Supplementary Fig. 4A, B) decreased TOPFlash activity, an effect that was rescued by reintroducing human CD44 (Supplementary Fig. 4C). Moreover, active β-catenin was undetectable by Western blot in *CD44* knockout HeLa cells (Supplementary Fig. 4A, B, D). We sought to follow CD44 influence on the accumulation of endogenous nuclear β-catenin after Wnt3a induction. To this end, we generated HeLa cells stably expressing a turnover-accelerated chromobody (Ub-R-BC1-CB) specifically recognizing soluble, hypophosphorylated, endogenous β-catenin [31, 32]. Confocal microscopy analysis of Ub-R-BC1-CB expressing cells showed that nuclear β-catenin in cells lacking was significantly reduced (Fig. 6a). This could be rescued by treating the cells with CHIR. Evaluation of full-cell accumulation of β-catenin by live fluorescent time-lapse microscopy showed equivalent results (Supplementary Fig. 5A–C). Moreover, the lack of *CD44* in the same cell line also led to reduced expression of Wnt target genes such as *AXIN2* and *NKD1*, which could be rescued by activation of the Wnt pathway with CHIR but not with Wnt3a (Supplementary Fig. 5D, E). This confirms our previous epistasis experiments that place the function of CD44 upstream of the β-catenin destruction complex [11].

In a previous study, we showed that CD44 forms a complex with LRP6 [11]. Here, we further explored this relationship of the endogenously expressed proteins by proximity ligation assays in the absence and presence of Wnt ligands. To this aim, HeLa cells were incubated with primary antibodies directed against LRP6 and CD44, and secondary antibodies coupled to PLA probes. Increased complex formation between CD44 and LRP6 was detected 15 min and 45 min after Wnt3a-conditioned medium (CM) treatment compared to control CM (Fig. 6b, c). To test the specificity of these results, we knocked out *CD44* by means of CRISPR/Cas9 (Supplementary Fig. 4A, B). In comparison to the parental HeLa cells, we observed very few signals in HeLa *CD44*^*-/-*^ cells. Similar results were observed for parental HeLa cells using only one primary antibody (Fig. 6c and Supplementary Fig. 6A). We then examined the relationship of CD44 with other members of the Wnt signalosome, such as DVL and AXIN. As shown in Fig. 6d, e, Wnt3a induction led to an increase in PLA signals as compared to CM-treated control cells. Very few events were observed in HeLa *CD44*^*−/−*^ cells (Supplementary Fig. 6C) for both interaction partners (Fig. 6d, e). Consistently, replacing one primary antibody with a suitable IgG isotype control gave no interaction signals (Supplementary Fig. 6B). Furthermore, co-immunoprecipitation experiments of DVL or AXIN with CD44 (Fig. 6f, g) further showed that CD44 forms part of the Wnt signalosome. In support of this, a sucrose gradient centrifugation assay demonstrated that phospho-LRP6 is shifted into higher fractions in Wnt stimulated cells, suggesting a Wnt-dependent assembly of multi-protein complexes (signalosomes) with greater molecular weight [33] (Supplementary Fig. 7). CD44 is present in these shifted fractions upon Wnt induction (Supplementary Fig. 7). The above observations are compatible with signalosome assemblies [33, 34] that include CD44.

### CD44 interacts directly with LRP6

**Fig. 7:**
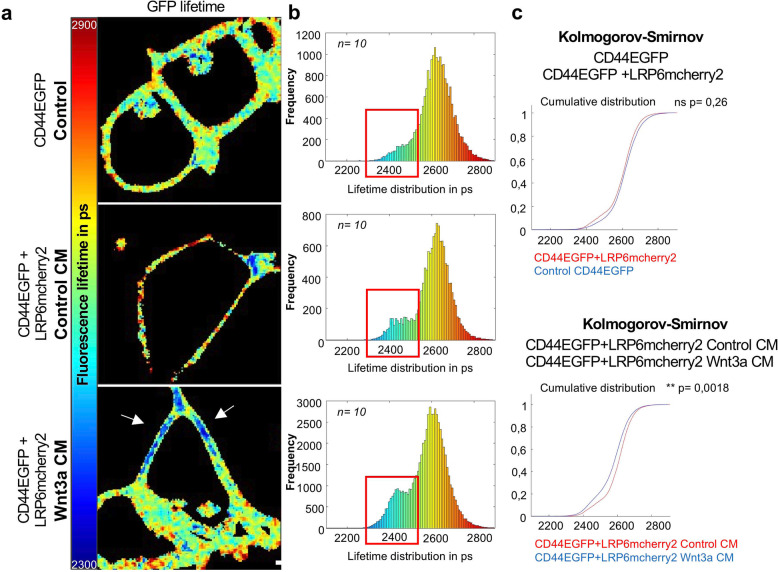
CD44 interacts directly with LRP6. **a** FRET-based FLIM analysis of HEK293T cells stably expressing CD44EGFP+LRP6mcherry2 or only CD44EGFP, induced with Wnt3a or ontrol CM 1 h before analysis. EGFP lifetime images according to the color scale. Scale bar: 10 μm. **b** EGFP lifetime distribution in the pixels of the recorded images of uninduced CD44EGFP cells or CD44EGFP+LRP6mcherry2 HEK293T cells treated with Wnt3a or Control CM (*n* = 10). Representative data of 1 out of 3 independent experiments. *n* = number of analyzed cells. **c** Kolmogorov–Smirnov test comparing the distributions of either the CD44EGFP and the CD44EGFP+LRP6mcherry2 cell lines or CD44EGFP+LRP6mcherry2 cell line treated with Wnt3a or Control CM. ns not significant.

Given that LRP6 and CD44 had the greater increase in PLA signals compared to CM-treated control cells, we decided to determine whether Wnt induction entails a direct interaction between CD44 and LRP6. We therefore performed FRET measurements on live cells between the two fusion proteins LRP6mcherry2 and CD44EGFP before and after induction of Wnt3a. Indeed, FRET considered as proof of direct contact between the two fusion proteins [35, 36], is only possible if the two fluorescent proteins are within 10 nm of each other [37]. We generated HEK293T cells stably expressing CD44EGFP and LRP6mcherry2 proteins by means of lentiviral transduction. The level of FRET was quantified at each pixel of the image by measuring the fluorescent lifetime of the donor EGFP dye using FLIM. In contrast to fluorescent intensity measurements, the fluorescent lifetimes do not depend on excitation intensity, nor fluorophore concentration and thus only depend on the existence of FRET [38, 39]. In cells co-expressing CD44EGFP and LRP6mcherry2 in the absence of Wnt3a, no significant change in the EGFP lifetime was observed as compared to cells expressing CD44EGFP alone (Fig. 7). In contrast, after Wnt3a induction, a significant contribution of a shorter fluorescence lifetime (around 2450 ps corresponding to a FRET of approximately 7.5%) was observed in a number of pixels at the plasma membrane (red rectangle). The obtained bimodal lifetime distribution significantly differed from the unimodal distribution observed before Wnt3a induction (*p*-value = 0.0018) (Fig. 7b, c) and suggested that Wnt3a ligand induction leads to a direct interaction between CD44 and LRP6 upon Wnt3a induction. Interestingly, the CD44EGFP shorter lifetime was not uniformly distributed over the membrane upon Wnt3a induction, but only appeared at discrete segments of the membrane (Fig. 7a) suggesting that CD44 and LRP6 preferentially interacted in specific membrane domains.

## Discussion

**Fig. 8:**
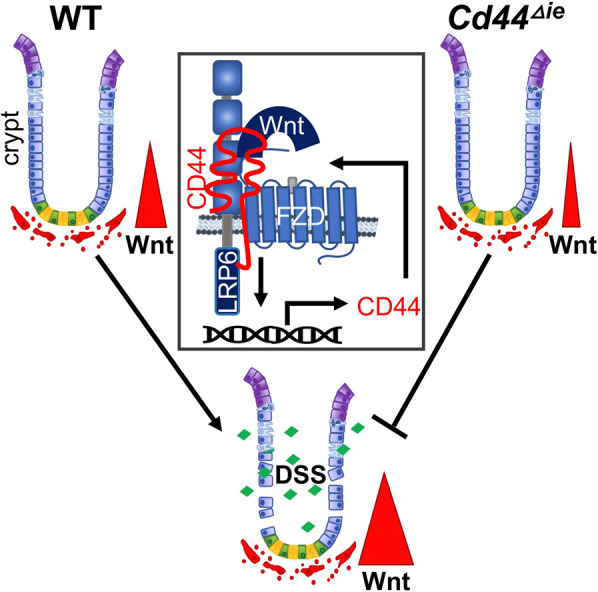
Graphical abstract. Since CD44 is a Wnt target gene and a positive modulator of signal transduction of the Wnt pathway, it establishes a positive feedback loop to amplify Wnt signaling in the intestine acting at the level of the signalosome. Removal of *Cd44* from the intestinal epithelium leads to decreased Wnt signaling still sufficient in homeostatic conditions but fails to increase Wnt activity as required in the regeneration of the intestinal epithelium after DSS damage.

We have presented a novel function of CD44 in the regulation of the Wnt pathway in the intestine. The removal of *Cd44* from the intestinal epithelium led to decreased Wnt signaling, which resulted in impaired regeneration of the intestine upon DSS treatment. Since CD44 is a Wnt target gene but also a positive modulator, CD44 is able to act as a positive feedback regulator to amplify Wnt signaling in the intestine (Fig. 8).

Loss-of-function of CD44 in the adult intestine is comparable to other models in which positive regulatory modules are affected. In knockout mouse models of RSPO3 or its receptor Lgr4 [40, 41], the levels of Wnt signaling are reduced. This generates mild phenotypes in homeostatic conditions but leads to severe consequences upon insults that require Wnt augmentation. As in our model, KO mice of RSPO3 in PdgfRα^+^ stromal cells [40] showed a decreased number of PCs during homeostasis, while being hypersensitive to DSS-induced colitis. Similarly, PCs were reduced in *lgr4*^*-/-*^ mice compared to control mice and intestinal inflammation was also more severe [41]. The DAI increased together with increased secretion of inflammatory cytokines and infiltration of immune cells, paralleling what we observed in the *Cd44* knockout in the intestinal epithelium. A difference between the RSPOs-Lgr4/5 and the CD44 modules is that the former depends on an additional external ligand input while the latter is directly activated by Wnt ligands in a positive feedback loop.

Wnt activity in the ISCs requires tight regulation. Given the high turnover rate of the intestinal epithelium, Wnt-signaling levels below a certain threshold can be lethal, as in mouse models of *Tcf4* conditional knockout [42] or DKK overexpression [43]. On the other hand, if this activity is too high it can lead to excessive crypt duplication, adenomas, and cancer [44]. This tight regulation is achieved, in part, by several molecular checkpoints and feedback loops, in which products of the pathway are part of the pathway itself. TCF4 and Wnt3a, for example, act as positive feedback regulators, increasing the pathway activity, while AXIN2 exerts the opposite effect. According to function and redundancy, the effect of a knockout of these molecules can have different impacts. Counteracting the lethal effect of *Tcf4* knockout, the replacement of Wnt3a by other Wnt ligands compensates its absence [45]. The case of CD44 is an intermediate one: since it is a positive regulator, its feedback function becomes apparent only when there is a necessity for Wnt signaling augmentation. Upon *Cd44* deletion in the intestinal epithelium, Wnt levels remain sufficient to ensure intestinal integrity with minor phenotypic changes, but the consequences of *Cd44* deletion observed during epithelial regeneration revealed its relevance and non-redundancy.

During intestinal epithelial regeneration following damage, the stem cell function needs to increase above its basal homeostatic levels, raising the Wnt minimal threshold [28, 40]. For example, in acute phases of DSS-induced colitis, stromal cells secrete more Wnt2b [46], while infiltrating macrophages produce other Wnt ligands [47–49]. However, increased availability of ligands can only amplify the pathway activity if other checkpoints of the process are released and the signal transduction is facilitated. Indeed, stromal cells also upregulate RSPO1 and Gremlin1 [46], stabilizing FZD on the cell membrane [6–8], while antagonizing BMP-signaling [50], respectively. CD44 positive regulatory function seems to be exerted at the membrane upon Wnt ligand binding to the tertiary complex. Coherently, CHIR stimulated Wnt pathway activation and target gene expression in *CD44*^*−/−*^ cells, whereas Wnt3a was unable to increase it. This was further confirmed by a reduced formation of cysts from *Cd44*^*−/−*^ SI organoids treated with Wnt3a.

Pleiotropic functions of CD44 as a co-receptor may have affected other pathways in the intestinal crypt. Kabiri and colleagues showed that Wnt signaling inhibits MAPK signaling. Therefore, upon Wnt downregulation, phospho-ERK accumulates in the crypt base [15]. This was not the case in our model presumably because activation of the MAPK signaling pathway by EGF or HGF [51, 52] is dependent on the co-receptor function of CD44 isoforms containing variant exon v6 [53, 54]. In support of this notion, until now the only functional evidence of CD44 in the intestinal epithelium was the co-receptor function of CD44v4–10 on MET signaling [51]. Of note, since we knocked out all CD44 isoforms, CD44v4–10 was also eliminated. However, in contrast to MET, the enhancement of Wnt signaling by CD44 is isoform-independent [11].

We observed a reduced number of PCs in *Cd44* knockout mice. This is compatible with the dependence on Wnt signaling for their maturation by the Wnt-induced MMP7/cryptidin program [2]. Lyz marks specified PCs independently of their maturation state. In Wnt loss-of-function models they can be specified but fail to mature [2]. Although stem/progenitor-cell gene and PC maturation programs are both Wnt-dependent, they are separately driven, inducing differentiation in the latter while maintaining stemness in the former [2]. These two opposite programs may have different Wnt activity thresholds. Here, the overall structure of the intestinal epithelium is maintained in the absence of CD44 and the stem cell number as assessed by OLFM4 staining [55] is not decreased. This observation is in contrast to Sato et al., 2011 [17] showing, in several mouse models, a reduced number of PCs accompanied by a decreased number of ISCs. However, our result agrees with other papers including van Es et al., 2018 [56] where the targeted elimination of PCs by diphtheria toxin, did not impact the ISC number. These discrepancies show that the consequence of PC deletion is still a matter of discussion. Since de-differentiation of PCs was not observed in homeostatic conditions [57], the decreased Lgr5 mRNA levels cannot be linked to an overall decreased capacity of PCs to de-differentiate. In regeneration, the defect observed after DSS-induced colitis is unlikely to be due to the reduced secretion of Wnt by PCs [40, 45] given the source of Wnt ligands from the stromal compartment in vivo. Another argument suggesting that the effects observed in intestinal regeneration upon *Cd44* deletion are linked to the critical function of CD44 in the Wnt signalosome at the membrane of intestinal stem/progenitor cells and are not connected to the reduced number of PCs comes from the reduced survival of colon organoids upon deletion of *Cd44*. Indeed, PCs are not present on the colon. The reduced β-catenin staining observed in colonic crypt bases, upon removal of *Cd44*, supports the notion of a direct effect of CD44 in the Wnt signalosome of ISCs of the SI, over an indirect effect of decreased PC number.

The relevance of the knockout of *Cd44* in the intestinal epithelium upon the requirement of augmented Wnt activity, having only a mild phenotype in homeostatic conditions depicts the potential of CD44 as a therapeutic target in pathologically augmented Wnt signaling, as in colorectal cancer.

## Methods

A resource table can be found in the Supplementary Material (Supplementary Table S1).

### Cell culture and generation of stable cell lines

HEK293T (ATCC Cat# CRL-3216, RRID: CVCL_0063); HeLa (ATCC Cat# CCL-2, RRID: CVCL_0030); L-Wnt-3A cells (ATCC Cat# CRL-2647, RRID: CVCL_0635) and L-Cells (ATCC CRL-2648, RRID: CVCL_4536) were obtained from ATCC (Wesel, Germany) and maintained in Dulbecco’s Modified Eagle Medium (DMEM) supplemented with 10% FBS and 1% PenStrep. NCI-H1703 cells (ATCC Cat# CRL-588, RRID: CVCL_1490) were obtained from ATCC (Wesel, Germany) and maintained in Roswell Park Memorial Institute (RPMI) 1640 medium supplemented with 10% FBS, 1 mM sodium pyruvate, and 1% PenStrep at 37 °C. All experiments were performed using mycoplasma-free cells.

Mouse Wnt3a-conditioned medium (CM) was produced from L-Cells stably transfected with Wnt3a. As a control conditioned medium (Co CM), medium from L-Cells was used. L-Wnt-3A cells or WT L-Cells were cultured in DMEM, Advanced DMEM/F12 or RPMI + 10% FBS for 4 days. The function of the Wnt3a CM to induce Wnt signaling in cells was tested by SuperTOPFlash assay. R-Spondin-conditioned medium (CM) was produced from HEK293T cells, stably transfected with R-Spondin (a kind gift from Dr. C. Kuo, (Stanford University, USA)). HEK-R-Spondin cells were cultured in Advanced DMEM/F12 for 5 days.

Stable overexpression cell lines were generated by producing lentiviral particles. Lentiviral expression plasmids (CD44gRNA (10 µg), CD44EGFP (5 µg), LRP6mcherry2 (10 µg)) encoding the gene of interest were transfected together with the envelope plasmid (vesicular stomatitis virus G protein, pVSV-G (2.8 μg)) and two packaging plasmids (pRSV-REV (2.5 μg), pMDLg/pRRE (5 μg)) into 80–90% confluent HEK293T cells cultured in DMEM or RPMI supplemented with 10% FBS using PromoFectin (PromoKine) according to the manufacturer´s protocol. After 6 h, the medium was replaced by serum-containing DMEM. After 24 h, the supernatant containing the virus particles was harvested, passed through a 0.45 μm filter, and transferred to the target cells, which were seeded the day before to reach 70% confluency. Transduced cells were selected by puromycin (10 µg/ml) or blasticidin (2 µg/ml) treatment for 3 weeks. The medium containing the antibiotics was changed every second day. The lentiviral vector carries the sgRNA sequence targeting *CD44 exon1* (DNA sequence sgRNA: 5’-TCCGTTCGCTCCGGACACCA-3’) upstream of the initial ATG. The sgRNA expression is driven by the human U6 promoter followed by the human eukaryotic translation elongation factor 1α1 short form (EFS) which drives the expression of Cas9 and the puromycin resistance linked by the T2A self-cleaving peptide. A polyclonal cell line has been selected via fluorescence-activated cell sorting (FACS) (see “Fluorescence-activated cell sorting” section). All cell types have not been authenticated.

### Experimental animals

#### Species used: *Mus musculus*

The *Cd44* floxed mouse (MGI:6199605) was crossed with the *VillinCreER*^*T2*^ mouse (MGI:3053826) in order to generate a *Cd44*^*fl/fl*^*;VillinCreER*^*T2*^ mouse, a tamoxifen-inducible knockout mouse model. The generation of *Cd44* floxed mice is described by Shatirishvili and colleagues [58]. All experiments were performed with male *Cd44*^*fl/fl*^;*VillinCreER*^*T2*^ backcrossed to C57BL/6 mice for 12 generations. Tamoxifen (50 mg/kg) (Sigma) diluted in peanut oil was injected IP to activate the inducible Cre in order to initiate the knockout of *Cd44* in the intestinal epithelium (*Cd44*^*Δie*^).

All animals used in this study were housed and maintained under specific pathogen-free conditions in 14/10-h light/dark cycles at 21 ± 1 °C and 55 ± 5% humidity in a facility approved by the Regierungspräsidium Karlsruhe. Food and water were provided ad libitum and weaning took place 21 days after birth. All animal procedures were performed in compliance with the German Animal Protection Act and with the approved guidelines of the Society of Laboratory Animals (GV-SOLAS) and of the Federation of Laboratory Animal Science Associations (FELASA). Experiments were authorized by the Regierungspräsidium Karlsruhe (approval number 35–9185/G-237/15).

### DSS treatment

DSS (MP Biomedicals) was dissolved at 2% in autoclaved tap water and provided for a time period of 5 days *ad libitum* as drinking water to mice. The mice were monitored in order to determine the DAI score including parameters for stool consistency, bleeding, and % of body weight loss. Scores are defined according to Kim et al. [22] with minor changes as follows: weight loss: 0 (none), 1 (1–5%), 2 (5–10%), 3 (10–20%), and 4 (>20%); stool consistency: 0 (normal), 2 (loose stool), and 4 (diarrhea); and bleeding: 0 (no bleeding), 2 (Hemoccult+), and 4 (gross bleeding). DAI was scored daily during the duration of the DSS treatment. The mice were sacrificed at day 4 post DSS treatment and after a regeneration period at day 19 post DSS. Their intestines were fixed in 4% paraformaldehyde (PFA) and subjected to histochemical analysis. Intestinal cross-sections were evaluated using the HCS, a score system that comprises five criteria, each of them assigned to scores between 0 and 3 (0 = no effect, 3 = very severe effect) [23]. The rated categories included mucosal and submucosal infiltration of immune cells, the severity of crypt damage, hyperplasia, and the overall severity of the tissue inflammation.

### Isolation and culturing of mouse intestinal organoids

Small intestines of 7–10 weeks old *Cd44*^*fl/fl*^*;VillinCreER*^*T2*^ mice were washed with PBS and opened longitudinally. Intestines were placed villi facing up. Villi were gently removed using a glass slide and the intestines were washed in PBS until no cell debris remained. The washed intestines were incubated with 2 mM EDTA for 30 min at 4 °C. Crypts were mechanically released from the surrounding tissue by shaking. The freed crypts were pelleted and passed through a 70 μm strainer. Isolated crypts were resuspended in Matrigel (Corning) and cultured in 500 μl complete IntestiCult™ Organoid Growth Medium (STEMCELL Technologies) based on the previously described standard ENR medium (Adv. DMEM F12 (Sigma), 50 ng/ml EGF (Peprotech), 100 ng/ml Noggin (Peprotech), 3 µm CHIR99021 (CHIR) (Selleckchem), B27 (Gibco), N2 (Gibco), 1 mM N-Acetyl cysteine (Sigma)) [16] in 24-well plates. 4-hydroxytamoxifen (4-OHT) (1 mM) (Sigma) or ethanol was added for 48 hours. Organoids were split by a mechanical breakup and dispersed crypts were reseeded every fifth day in a 1:2 ratio for 2 passages. Organoids were treated with CHIR (15 µM) or DMSO in IntestiCult™ Organoid Growth Medium (STEMCELL Technologies) or in ENR supplemented with 50% Wnt3a CM and 20% R-Spondin CM or 5% R-Spondin CM only.

### Isolation and culturing of mouse colon organoids

The colon (5–7 cm of the proximal part) of 5–7-week-old *Cd44*^*fl/fl*^*;VillinCreER*^*T2*^ mice was flushed with cold PBS and opened longitudinally. In order to remove fecal matter and mucus, a glass slide was used to gently scrape the lumen. The colon was washed in cold PBS until the supernatant no longer contained any visible debris before it was cut in 5 mm pieces which were vigorously pipetted up and down (15×) in 5 mM EDTA-PBS. Fragments were incubated in fresh 5 mM EDTA-PBS (10 ml) for 15 min on a benchtop roller at 4 °C and afterwards vigorously triturated by pipetting up and down (15×) and washed using PBS (2×). Basal Medium containing 500 U/ml Collagenase A was added and fragments were once again pipetted up and down (5x) before incubation in a water bath at 37 °C for 30 min. Crypts were mechanically released from the surrounding tissue by vigorously triturating the fragments (10×) in 10 ml PBS. Basal Media containing 15 U/ml DNase I (10 ml) was added and freed crypts were passed through a 100 μm strainer and additionally through a 70 μm filter. Crypts were centrifuged at 300 × *g* for 5 min and the pellet was seeded in Matrigel (Corning) and cultured in 500 µl colon organoid growth medium (IntestiCult™ supplemented with 70 µl Wnt3a CM). Organoids were split by mechanical breakup after 5–7 days in a 1:2 ratio.

### Immunohistochemistry

Mouse tissue was fixed in 4% PFA overnight and embedded in paraffin. 4 µm sections were deparaffinized with Xylene and hydrated in 100% and 70% ethanol on slides. The antigen retrieval technique was performed according to the HIER protocol. Slides were covered with 10 mM citrate buffer (pH 6) and boiled 2 × 5 min. The slides were cooled in the buffer and incubated with 3% hydrogen peroxide for 12 min to inactivate endogenous peroxidases and then washed with PBS-T (0.1% Tween-20 in PBS). Slides were blocked in 5% FBS in PBS-T for one hour at room temperature (RT) and then incubated with the primary antibody overnight at 4 °C in blocking buffer. IHC was performed using antibodies directed against Cleaved caspase-3 (1:500; Cell Signaling Cat# 9661T, RRID: AB_234118), F4/80 (1:200; Cell Signaling Cat# 70076S, RRID: AB_2799771), Ki67 (1:200; abcam Cat# ab15580, RRID: AB_443209) and OLFM4 (D6Y5A) (1:400; Cell Signaling Cat# 39141S, RRID: AB_2650511). After washing in PBS-T, slides were incubated for one hour with the following secondary antibody: Goat anti-rabbit biotinylated secondary antibody, (1:200; Dako Cat# E0432, RRID: AB_2313609). Biotinylated HRP was pre-incubated with avidin to form large avidin-biotin-enzyme complexes (StreptABC-complex). Slides were washed with PBS-T and incubated with StreptABC-complexes for one hour at RT. After washing with PBS-T the slides were incubated with the substrate 3,3′-Diaminobenzidine (DAB) (Vector Laboratories) for 10 and 15 min. Before a subsequent counterstain of the nuclei, the slices were washed in tap water. The slides were incubated for 1 min in hematoxylin and 1 min in running tap water followed by rehydration steps by incubation in 70% and 100% ethanol followed by Xylene. Sections were mounted with Eukitt Quick-hardening mounting medium and a coverslip.

The staining against β-catenin (1:500; BD Biosciences Cat# 610154, RRID: AB_397555), was performed using the EnVision+ System-HRP (Dako) protocol. Prior to the addition of the primary antibody, the slices were incubated for 10 min with a power block solution, blocking the peroxidases. After washing with PBS-T, the primary antibody (β-catenin 1:500) was added to the slides and incubated overnight at 4 °C. Slices were washed to remove the primary antibody and then incubated with Rabbit EnVision+System-HRP (Dako) or Mouse EnVision+System-HRP (Dako) for 30 min. Sections were rinsed in PBS-T before applying DAB. Slices were washed and counterstained with hematoxylin, rehydrated, and mounted as described above.

Immunohistochemistry staining against β-catenin of Swiss-roll sections of the colon of two *Cd44*^*+/+*^ and three *Cd44*^*Δie*^ mice after tamoxifen induction were scanned and the images were analyzed using the Aperio Imagescope – Pathology Slide Viewing Software (Leica Biosystems). Frames of all complete crypts (with a middle sagittal cut, recognizing its lumen) were transformed into TIFF format and analyzed, using Integrated Density (IntDen) analysis of regions of interest (ROIs), drawn by hand on the crypt bottom (including 10–12 cells) using ImageJ (NIH, USA). Since the staining intensities were uneven between samples and within samples, and considering an intense membrane β-catenin staining, 2 ROIs of transit-amplifying cells were taken on each crypt and the average value of their IntDens was subtracted to the IntDen of the crypt bottom.

The Alcian blue and PAS staining was performed after a deparaffinization and rehydration step following a standard staining protocol of 15 min Alcian blue (1% Alcian blue in 3% acetic acid in distilled water), 2 min running tab water, 5 min periodic acid (0.5% periodic acid in distilled water), 5 min tab water, 10 min Schiff’s reagent, 2 min running tap water followed by a hematoxylin staining.

### Immunofluorescence staining of paraffin sections and mouse intestinal organoids

Paraffin-embedded tissues were prepared as described for IHC up to the peroxidase blocking step. Sections were treated with a blocking solution containing 5% FBS in PBS-T for 1 h at RT and then incubated with the primary antibody overnight at 4 °C in blocking buffer. Immunofluorescence was performed using antibodies directed against lysozyme (1:100; Thermo Fischer Cat# PA5–16668, RRID: AB_10984852), myeloperoxidase (1:200; abcam Cat# Ab75358, RRID: AB_2139308), CD44 (IM7) (2.5 µg/ml; BD Biosciences Cat# 550538, RRID: AB_393732), chromagranin A (1:200; abcam Cat# Ab15160, RRID: AB_301704). After washing in PBS-T, slides were incubated for 1 h with the following secondary antibodies and DAPI (Sigma-Aldrich Cat# D21490) at RT: Donkey anti-Rabbit IgG secondary Antibody; Alexa Fluor® 488 conjugate (1:500; Invitrogen Cat# A21206, RRID: AB_2535792), Goat anti-Rat IgG secondary Antibody; Alexa Fluor® 488 conjugate (1:500; Thermo Fisher Cat# A11006, RRID: AB_2534074), Goat anti-Rabbit IgG secondary Antibody; Alexa Fluor® 546 conjugate (1:500; Thermo Fisher Cat# A11071, RRID: AB_2534115).

Mouse intestinal organoids were isolated from Matrigel domes using 4 °C cell recovery solution (Corning). The supernatant was removed and the organoids were fixed in 2% PFA supplemented with 0.1% glutaraldehyde for one hour at RT. Following two washing steps with PBS, the organoids were permeabilized in 0.1% Triton X-100 for 30 min. Organoids were treated with a blocking solution containing 5% FBS in PBS for 30 min at RT. IF was performed using an antibody directed against CD44 (2.5 µg/ml) overnight at 4 °C on an orbital shaker. After washing with PBS-T, organoids were incubated with the Goat anti-rat IgG secondary antibody, Alexa Fluor® 488 conjugate (1:500; Thermo Fisher Cat# A11006, RRID: AB_2534074), for 2 hours at RT. Organoids were counterstained with DAPI (1 µg/ml).

Tissue sections or organoids were washed in PBS-T, mounted with fluorescence mounting medium (Dako), and imaged with a Zeiss LSM 800 confocal microscope. Images were processed using Fiji (U.S. NIH).

### RNA in situ hybridization

In situ hybridization analysis was performed by using RNAscope 2.5 LS (Brown) detection kit (Advanced Cell Diagnostics) on a Leica Bond Rx autostainer according to the manufacturer’s instructions. Detection of the expression of *Axin2* and *Lgr5* was analyzed. Positive staining was identified as brown, punctate dots. Crypts were encircled manually and the positive staining area for each crypt was measured via Fiji. A threshold was set to mark the area of positive staining only. This threshold was maintained for the analysis of the entire image. Intestinal sections were counterstained with hematoxylin to visualize the nuclei. *Ppib* was used as a positive control.

### Fluorescence-activated cell sorting

FACS was performed with a FACSAria^™^ Flow Cytometer from BD Biosciences. In order to select a polyclonal cell subline in which CD44 is absent, cells were detached using Accutase and collected in serum-containing medium. A total amount of 1 × 10^7^ cells was used for sorting. Cells were resuspended in 1 ml (100 µl/10^6^ cells) FACS buffer (2% FBS; 2 mM EDTA; PBS) and incubated with human-specific Fc-block (2 µl/10^6^ cells) for 45 min on ice. Surface CD44 was labeled with the anti-CD44 antibody IM7 conjugated to PE or APC/Cy7 (IM7-PE; 1:100, IM7-APC-Cy7, 1:100) and incubated for 15 min at RT in the dark. A corresponding isotype control (PE Rat IgG2bκ, Biolegend Cat# 400607, RRID: AB_326551, APC/Cy7 Rat IgG2bκ; Biolegend Cat# 400623. RRID: AB_326565) was used. Cells were washed with FACS buffer and resuspended in 500 µl PBS. Data was analyzed using the FlowJo software (license number M11c3c353YH92SCS). Successful sorting was verified via FACS analysis. The expression of CD44 in WT vs. knockout cells was compared.

### TOP-Flash reporter assay

HEK293T cells (1.5 × 10^4^/well) were seeded in a 96-well plate and transfected with SuperTOPFlash reporter (20 ng), TK-Renilla (5 ng) vector (for normalization purposes), and the respective concentrations of hCD44 (50 ng, 100 ng) using PromoFectin (PromoKine) transfection reagent according to the manufacturer’s protocol. Each transfection sample was adjusted to 125 ng DNA/transfection with pcDNA3.1 empty vector. Cells were induced with Wnt3a CM 24 hours after DNA transfection. Cells were lysed 48 h post-transfection with 1× Passive Lysis Buffer (Promega). Luciferase activity was measured using a Luminescence counter 1420 (PerkinElmer, Rodgau, Germany). In addition to the reporter assay, transfected cells were subjected to Western blot analysis.

### Chromobodies

HeLa (ATCC) and HeLa_CD44^−/−^ cells were allowed to grow to 70% confluency before the expression vector encoding the Ubi-R-BC1-eGFP2 construct [31, 32] was transfected using Lipofectamine 2000 (Thermo-Fisher Scientific) according to the manufacturer’s protocol. 24 h post-transfection, cells were subjected to 0.5 mg/ml G418 (Roth) for a selection period of 3 weeks. Cells were induced using Wnt3a CM for 24 h and eGFP positive cells were FACS sorted. Wnt pathway activation was induced either by Wnt3a CM or by 10 µM CHIR. Cells were analyzed regarding the level of Ub-R-BC1-eGFP2 expression after induction via live-cell imaging (Incucyte®, Sartorius) or fixed 24 h after induction. For live-cell imaging, 0.3 × 10^4^ cells were seeded in a 96-well plate and induced the day after. The increase in fluorescent intensity was measured every 2 h up to 26 h. For the experiment using fixed cells, 0.6 × 10^5^ cells were seeded on a glass slide in a 12-well plate. Wnt pathway activation was induced by Wnt3a CM or CHIR the day after seeding. 24 h after induction, cells were washed with PBS and fixed in 2% PFA (10 min at 4 °C), followed by washing steps. Before mounting with fluorescence mounting medium (Dako), cells were incubated with DAPI (1:10,000 in PBS, 10 min at RT in the dark).

#### Image segmentation and analysis

For quantification (live cell condition), an Incucyte® software mask was applied that differentiates cells from the background (plastic) in order to define the cell area in which the fluorescent intensity for each picture is measured. The fluorescence values were normalized to a respective non-treated control and the mean of the overall fluorescence of each picture was plotted against time.

For quantification (fixed condition), the mean fluorescence in a defined (segmented) area of interest (nucleus) was determined. Using Fiji, a nuclear and background mask was generated. The area of the defined nuclei was increased in all directions with a certain size (minimal and maximal width) and shape parameters in order to define the cytoplasmic parts of the cells. This step was followed by an inversion in order to define the segmentation mask of the background fluorescence. The average fluorescence intensities of the chromobodies in the nuclear mask was subtracted from the background fluorescence, defined by the segmentation mask.

### Western blotting

Cells were lysed in 1% Triton lysis buffer (1% Triton X-100, 50 mM Tris-HCl (pH 7.4), 150 mM NaCl, 25 mM NaF, 5 mM sodium orthovanadate (Na_3_VO_4_), 0.1% NP-40, 1 mM EDTA pH 8.0, 1 M EGTA, protease inhibitor cocktail (Roche), pH7) and boiled in 4× Laemmli Sample Buffer (Bio-Rad) supplemented with 2-Mercaptoethanol (Roth). Lysates were subjected to standard SDS-PAGE and Western blotting procedures using primary antibodies directed to CD44 (1:1000; Hermes-3), LRP6 (1:1000; Cell Signaling Cat# 2560 S, RRID: AB_2139329), p-LRP6 (1:1000; Cell Signaling Cat# 2568 S, RRID: AB_2139327), DVL2 (1:1000; Cell Signaling Cat#3216, RRID: AB_2093338), Vinculin (1:1000; Cell Signaling Cat# E1E9V, RRID: AB_2728768) and anti-Rabbit-HRP (1:2000; Dako Cat# P0448, RRID: AB_2617138), anti-Mouse-HRP secondary antibodies (1:2000; Dako Cat# P0447, RRID: AB_2617137). Signal detection was performed by using the enhanced chemiluminescence system (ECL; Thermo Fisher, Schwerte, Germany) and the ChemiDoc^TM^ MP Imaging System (Bio-Rad). If necessary, membrane-bound antibodies were removed by incubation with stripping solution for 30 min at 55 °C (62.5 mM Tris, pH 6.8; 2% SDS; 0.8% DTT) and incubated with loading control antibodies.

Uncropped western blots can be found in the Supplementary Material.

### Co-immunoprecipitation of endogenous proteins

HeLa cells (1 × 10^6^) were seeded in 10 cm dishes and incubated for the indicated time points with Wnt3a CM. Cells were washed with ice-cold PBS on ice and lysed in 1% Triton lysis buffer for 30 min. Clear lysates (12.000 rpm for 15 min at 4 °C) were supplemented with CD44 antibodies (Hermes-3, 3 µg) or AXIN (1:100; Cell Signaling Cat# 2087, RRID: AB_2274550) and incubated at 4 °C overnight. Protein G agarose beads (Merck Cat#16–266) or mouse/rabbit-IgG sepharose bead conjugates (IgG-control) (Cell Signaling Cat# 3420, RRID:AB_1549744; Cell Signaling Cat# 6990, RRID: AB_10828434) were added to the lysates for 3 hours the day after. Precipitates were washed in lysis buffer and boiled in 4x Laemmli Sample Buffer (Bio-Rad) supplemented with 2-Mercaptoethanol (Roth). Samples were subjected to Western blotting analysis.

### Proximity ligation assay

0.6 × 10^5^ HeLa or HeLa^−/−^ cells were seeded on glass slides in a 12-well plate. Cells were fixed in 4% PFA for 10 min at 4 °C. After fixation, cells were washed with PBS and permeabilized (0.1% Triton X-100) for 15 min at RT. After washing with PBS, cells were incubated with Duolink® Blocking Solution for 45 min at 37 °C. Primary antibodies directed against CD44-ICD (1:200; Trans Genic Inc. Cat# KO601, RRID: AB_2833239), LRP6 (1:100; abcam Cat# ab75358, RRID: AB_2139308), DVL2 (1:100; LSBio Cat# LS-C340131, RRID: AB_2833240), AXIN (1:100; antikörper-online.de) or IgG control (Cell Signaling Cat# 011–01, RRID: AB_1550038, Cell Signaling Cat# 5415 S, RRID: AB_10829607) were diluted in Duolink® Antibody Diluent and incubated overnight at 4 °C in blocking solution. The next steps were performed according to the manufacturer’s Duolink^®^ Proximity Ligation Assay (Sigma) protocol. Cells were imaged with a Zeiss LSM 800 confocal microscope. Images were processed using Fiji (U.S. NIH). The number of green fluorescent dots of around 80 cells per condition was counted for each of 3 independent experiments.

### RNA isolation and quantitative real-time PCR

For mice samples, approximately 2 cm of the colon was isolated, supplemented with Trifast peqGOLD (Peqlab), and homogenized with an electrical tissue homogenizer. After centrifugation, an equal volume of 70% ethanol was added to the supernatant and RNA was extracted using the QIAGEN RN easy kit, following the manufacturer’s instructions. Prior to cDNA synthesis, *DNase I* was added to the isolated RNA (30 min at 37 °C). Next, DNase stop solution (Promega) containing EDTA (10 min at 65 °C) and random primers were added (1 µl) (5 min at 70 °C). cDNA synthesis reactions (10 µl) were prepared of 4 µL 5× MLVRT buffer (Promega), 2 µl dNTPs and 1 µl reverse transcriptase (MLVRT; Promega). Samples were incubated 10 min at 25 °C followed by 60 min incubation at 42 °C. The qPCR reactions (20 µl) consisted of 10 µl 2× GoTaq® qPCR Master Mix (Promega), 0.5 mM reverse and forward primers, and 2 µl cDNA. The qPCR was performed according to the manufacturer’s protocol on the StepOnePlus™ Real-Time PCR system (Applied Biosystems). *Gapdh* and *β-Actin* were used as reference genes for relative quantification according to the Litvak method (ΔΔCt). Primers were designed using the Primer3 open software (http://primer3.ut.ee) upon NCBI-database transcript sequences, and are detailed in Supplementary Table 2.

### Fluorescence lifetime imaging microscopy

The potential binding partners LRP6 and CD44 were labeled with spectrally distinct fluorophores at the N-terminal part. LRP6 was tagged with mCherry2, CD44 was tagged with EGFP. Both proteins are stably expressed in lentivirally transduced HEK293T cells. Time-correlated single-photon counting FLIM experiments were performed on a home-built multiphoton microscope based on an inverted microscope (IX70, Olympus, Tokyo, Japan) as described in Clamme et al. [59] and Azoulay et al. [60]. Fluorescence of EGFP was excited at 930 nm with a Ti-Sapphire laser (Tsunami, Spectra Physics) and detected through an avalanche photodiode (APD SPCM-AQR-14-FC, Perkin Elmer). The fluorophore mCherry2 was excited by the emitted light from GFP. Imaging was implemented by using a laser scanning system with two fast galvo mirrors (Model 6210, Cambridge Technology, Bedford, MA, US) which operate in the descanned fluorescence collection mode. The fluorescence was led to a fiber-coupled avalanche photo diode connected to a time-correlated single-photon counting module (SPC830, Becker&Hickl, Berlin, Germany), which works in a reversed start-stop mode. Photon statistics was used to define the fluorescence decays. The excitation wavelength at 930 nm was provided by a mode-locked Ti-Sapphire laser. Afterward, the data were analyzed by FLIMfit software (Photonics Group of the Physics Department at Imperial College London), while the statistical analysis of the lifetime distribution was performed with a home-made matlab program. FRET efficiency reflecting the distance between the two chromophores was calculated according to:

$$Ea = 1 - \frac{{\tau _{{\rm{DA}}}}}{{\tau _{\rm{D}}}}$$

where *τ*_DA_ corresponds to the lifetime of the donor in the presence of the acceptor and *τ*_D_ reflects the lifetime of the donor in the absence of the acceptor.

### Sucrose gradient sedimentation

HEK293T cells were seeded in 10 cm culture plates (2 × 10^6^) and transfected with CD44s (1.5 µg), LRP6 (2 µg), and MesD (500 ng) about 20 h later. The total amount of transfected DNA was filled up to 4 μg with a pcDNA3.1 empty vector. The transfection was performed using PromoFectin (PromoKine) according to the manufacturer’s protocol. 48 h after DNA transfection, cells were treated for 3 h with Wnt3a CM or control CM. Cells were lysed in 500 µl 1% Triton lysis buffer for 20 min on ice. The clear cell lysate was layered on top of a 5 ml 15–40% continuous OptiPrep (Sigma) (OptiPrep solution diluted in 0.02% Triton lysis buffer) gradient. Gradient centrifugation was performed for 4 h at 45,000 × *g* and 4 °C using a SW50.1 rotor in an ultracentrifuge (Beckman). After centrifugation, fractions were collected from the bottom of the tube using a peristaltic pump and analyzed by SDS-PAGE and Western blot.

### Patient survival and correlation matrix data

Patient data were obtained from public data and analyzed by the Kaplan–Meier plotter tool (GEPIA2 prognostic database) as described elsewhere [61].

The cohort was separated by the median of corresponding gene expression (“High” and “Low” respectively). Analysis was performed for relapse-free survival in the cohort of Crohn’s disease patients (*n* = 112) (GSE137344). The correlation matrix analysis by Corrplot package was realized from gene expression measured by RNAseq on ileal biopsies of 74 Crohn’s disease patients (GSE171244). The graphical representation was generated using R package corrplot (https://github.com/taiyun/corrplot, Taiyn and Simko). The multiple testing corrections were performed using BH method [62].

### Quantification and statistical analysis

Image analysis was performed using Fiji (RRID:SCR_002285). Statistical analysis was conducted using GraphPad Prism 9.0.0 (GraphPad, RRID:SCR_002798) and Microsoft Excel V16.31 (Microsoft, RRID:SCR_016137). Data are represented as mean values ± standard deviation (SD) or standard error (SE) from at least three independent experiments. Detailed sample size, biological or technical replicates, independent experiments, and statistical tests used for each experiment are indicated in the figure legends. Mean values of quantitative variables between two independent groups were compared using a Student’s *t* test if datasets had a normal distribution (Shapiro–Wilk test) or using Mann–Whitney *U*-test for non-normal distributions. We accepted an *α* = 0.05. *p* values are indicated in the corresponding graphs. To compare distributions, the Kolmogorov–Smirnov test was used. *p* values < 0.05 were considered statistically significant.

## Supplementary information

Supplementary Figure Legends

Supplementary Material

Supplementary Figure 1

Supplementary Figure 2

Supplementary Figure 3

Supplementary Figure 4

Supplementary Figure 5

Supplementary Figure 6

Supplementary Figure 7

Original Data File

aj-checklist

Agreement author list

declaration of contributions to article

Cover letter with competing interest statement

## Data Availability

The data can be made available upon reasonable request to the corresponding author at the following address: Véronique Orian-Rousseau, Karlsruhe Institute of Technology, Institute of Biological and Chemical Systems – Functional Molecular Systems, Karlsruhe, Campus North, Postfach 3640, 76021 Karlsruhe, Germany. e-mail: veronique.orian-rousseau@kit.edu.
