# Supplementary material for: Wnt signaling is boosted during intestinal regeneration by a CD44-positive feedback loop
Source: Cell Death Dis. 2022 Feb 21;13(2):168. doi: 10.1038/s41419-022-04607-0 (PMC8861016; doi:10.1038/s41419-022-04607-0)
Supplement: Supplementary file 1 — Supplementary Figure Legends [file 41419_2022_4607_MOESM1_ESM.docx]

**Supplementary Figure legends**

**Supplementary Figure 1. Related to Figure 1. Intestinal crypt-villus structure is conserved upon epithelial deletion of *Cd44***

**(A)** Gross morphology of colon (a-d) and SI (e-f) from *Cd44^Δie^* and *Cd44^+/+^* mice 18 and 33 days post tamoxifen treatment. Representative H&E staining in various magnifications, Scale bar: 200 µm (a’-d’ is an enlargement of a-d).

**(B)** Representative confocal images of SI (a-d) and colon (e-h) sections stained by the panCD44 antibody IM7 (green). Nuclei were counterstained with DAPI (blue) (a’-h’). Scale bar: 50 µm. TAM: Tamoxifen, SI: small intestine. Arrowheads: *lamina propria*

**Supplementary Figure 2. Related to Figure 1. Consequences in the intestinal epithelium upon *Cd44* deletion**

**(A)** Representative AB/PAS staining of sections of the SI from *Cd44^+/+^* and *Cd44^Δie^* mice. Goblet cells (GCs) were stained in blue. Scale bar: 50 μm.

**(B)** Quantification of stained GCs. n= number of villi.

**(C)** Representative confocal image of sections of the SI of *Cd44^+/+^* and *Cd44^Δie^* mice stained for ChgA (green). Scale bar: 50 μm. Arrowhead: ChgA^+^ EECs

**(D)** Quantification of the number of ChgA stained cells. n= number of villi.

**(E)** Representative IHC images of cleaved caspase-3 following *Cd44* deletion on sections of the SI of *Cd44^Δie^* compared to *Cd44^+/+^* mice. Scale bar: 50 μm.

**(F)** Quantitative analysis of the average number of cleaved caspase-3 positive cells per crypt. n= number of crypts.

**(G)** Representative images of IHC staining using antibodies against Ki67. SI of *Cd44^Δie^* compared to *Cd44^+/+^* mice. Scale bar: 50 μm.

**(H)** Quantitative analysis of the average number of Ki67 positive cells per crypt. n= number of crypts.

**(I)** β-catenin IHC staining in colonic crypts. Scale bar: 50 μm.

**(J)** Quantitative analysis of the β-catenin IHC staining in colonic crypts.

**(K)** Representative images of IHC staining using antibodies against p-ERK. SI of *Cd44^Δie^* compared to *Cd44^+/+^* mice. Scale bar: 50 μm.

Data are means ± S.E*.* Where indicated, Student’s t-test. Except S2D and S2F Mann Whitney U-test. ns= not significant, ***p < 0.001. N= Number of mice, ChgA: chromogranin A.

**Supplementary Figure 3. Related to Figure 2.** ***Ex vivo* knockout of *Cd44* in small intestinal and colon organoids**

**(A)** Small intestinal crypts were treated with 0.5 μM 4-OHT for 24 hours and derived intestinal organoids were examined by IF using antibodies against all CD44 isoforms (green). Nuclei were counterstained with DAPI (blue). Organoids were cultured in Matrigel and regular ENR medium, Scale bar: 50 µm.
**(B)** Relative colon organoid number along passage of *Cd44^fl/fl^;VillinCreER^T2^* organoids cultured with or without 4-OHT treatment. N= 3 mice.
**(C)** Representative images of **(B)** Scale bar: 300 µm
4-OHT: 4-hydroxytamoxifen.

**Supplementary Figure 4. Related to Figure 6. Wnt signaling activity can be rescued by CD44 re-expression**

**(A)** Flow cytometry analysis of CD44 expression in *CD44^-/-^* HeLa and NCI-H1703 cells compared to corresponding controls.

**(B)** Western blot analysis of *CD44^-/-^* HeLa and NCI-H1703 samples and corresponding controls using antibodies against all CD44 isoforms.

**(C)** TOPFlash analysis in NCI-H1703 WT, NCI-H1703 *CD44^-/-^* and NCI-H1703 *CD44^-/-^* cells transfected with 300 ng hCD44 and treated with Wnt3a CM. Error bars, ± SE.

Where indicated, Student’s t test; *p < 0.05. N= Number of biological replicates.

**(D)** Western blot analysis of Co-CM and Wnt3a CM-treated Hela and HeLa *CD44^-/-^* cells using antibodies against β-catenin, active β-catenin and CD44.

**Supplementary Figure 5. Related to Figure 6. β-catenin stabilization is reduced in *CD44* knockout cells**

**(A)** Fluorescence images of HeLa cells stably expressing the β-catenin-specific chromobody (CB) BC1-TagGFP2 (HeLa_BC1-TagGFP2) treated with either 10 µM CHIR or Wnt3a CM. Scale bar: 50 µm.

**(B)** Fluorescence images of HeLa *CD44^-/-^* cells stably expressing the β-catenin-specific chromobody (CB) BC1-TagGFP2 (HeLa *CD44^-/-^*_BC1-TagGFP2) treated with either 10 µM CHIR or Wnt3a CM. Scale bar: 50 µm.

**(C)** Quantification of the fluorescence intensity of HeLa_BC1-TagGFP2 and HeLa CD44^-/-^_BC1-TagGFP2 cells stably expressing the chromobody against β-catenin. The fluorescence intensity of the cells treated with either Wnt3a CM or CHIR was analyzed and plotted against time with the Incucyte software (N= 3, n= 256). Error bars, ± SE. n= number of analyzed cells.

**(D+E)** qPCR of the Wnt target genes *AXIN2* and *NKD1* in HeLa_BC1-TagGFP2 and HeLa CD44^-/-^_BC1-TagGFP2 cells. N= 3. Error bars, ± SE
Student’s t test; *p < 0.05, ns= not significant. N= Number of technical replicates

**Supplementary Figure 6. Related to Fig. 6. Interaction of CD44 with proteins of the signalosome is specific**

**(A)** PLA control experiments: HeLa cells incubated with only one primary antibody and the suitable IgG control of the second primary antibody; HeLa *CD44^-/-^* cells incubated with LRP6 and CD44 primary antibodies and both PLA probes (N= 1), Scale bar: 10 μm.

**(B)** PLA control experiments: HeLa cells incubated with only one primary antibody, the suitable IgG control of the second primary antibody and both PLA probes (N= 1), Scale bar: 10 μm.

**(C)** PLA control experiments: HeLa *CD44^-/-^* cells were incubated with DVL or AXIN and CD44 primary antibody as well as both PLA probes (N= 1), Scale bar: 10 μm.

N= Number of technical replicates

**Supplementary Figure 7. Related to Figure 6. CD44 is shifted into fractions with higher density upon Wnt3a induction**

Sucrose gradient sedimentation analysis of Triton X-100 lysates obtained from HEK293T cells induced with Wnt3a CM or control CM for 3 hours. HEK293T cells were transfected with CD44, LRP6 and MesD 48 hours before lysis. Proteins were separated in a continuous 15% to 40% OptiPrep gradient by ultracentrifugation (density increases to the right). Pooled fractions were analyzed by Western blot using antibodies against total LRP6, phosphorylated LRP6 (Ser1490) and all CD44 isoforms.
