# Supplementary material for: Wnt signaling is boosted during intestinal regeneration by a CD44-positive feedback loop
Source: Cell Death Dis. 2022 Feb 21;13(2):168. doi: 10.1038/s41419-022-04607-0 (PMC8861016; doi:10.1038/s41419-022-04607-0)
Supplement: Supplementary file 2 — Supplementary Material [file 41419_2022_4607_MOESM2_ESM.docx]

**Supplementary Material**

***Supplementary Table S1:* Resource table**

| Reagent or Resource | Source | Identifier |
| --- | --- | --- |
| **Antibodies** | | |
| APC/Cy7 anti-mouse/human CD44 (IM7) | Biolegend | Cat#103027, RRID: AB_830784 |
| AXIN | Cell Signaling | Cat#2087, RRID: AB_2274550 |
| AXIN1 | Antikörper-online.de | Cat#(AA 643-740) |
| Chromogranin A | abcam | Cat#ab15160, RRID:AB_301704 |
| CD44 ICD | Trans Genic Inc. | Cat#KO601, RRID:AB_2833239 |
| Cleaved caspase-3 | Cell Signaling | Cat#9661T, RRID: AB_2341188 |
| DVL2 | LSBio | Cat#LS-C340131, RRID:AB_2833240 |
| DVL2 | Cell Signaling | Cat#3216, RRID:AB_2093338 |
| F4/80 | Cell Signaling | Cat#70076S, RRID: AB_2799771 |
| Hermes 3 (human CD44 pan) | Gift from S. Jalkannen (Turku, Finland) |  |
| Ki67 (ab15580) | abcam | Cat# ab15580,RRID: AB_443209 |
| Lysozyme | Thermo Fisher | Cat#PA5-16668, RRID: AB_10984852 |
| LRP6 (C5C7) | Cell Signaling | Cat#2560S, RRID: AB_2139329 |
| LRP6 (ab24386) | abcam | Cat#Ab24386, RRID: AB_2139309 |
| LRP6 (ab75358) | abcam | Cat#Ab75358, RRID:  AB_ 2139308 |
| IM7 (CD44 pan) | BD Biosciences | Cat# 550538, RRID: AB_393732 |
| Myeloperoxidase | abcam | Cat#Ab9535, RRID: AB_307322 |
| OLFM4 (D6Y5A) | Cell Signaling | Cat#39141S, RRID: AB_2650511 |
| PE-anti-mouse/human CD44 (IM7) | Biolegend | Cat#103008, RRID: AB_312959 |
| Phospho-LRP6 (Ser1490) | Cell Signaling | Cat#2568S, RRID: AB_2139327 |
| Vinculin | Cell Signaling | Cat#E1E9V, RRID: AB_2728768 |
| β-catenin | BD Biosciences | Cat#610154, RRID: AB_397555 |
| Non-phospho (Active) β-catenin | Cell Signaling | Cat# 8814, RRID: AB_11127203 |
| Anti-mouse HRP-conjugated (Envision) | Dako | Cat#K4001, RRID: AB_2827819 |
| Anti-Phycoerythrin-Microbeads | Miltenyi Biotec | Cat#130-048-80, RRID: AB_244373 |
| Donkey anti-rabbit Alexa 488 | Invitrogen | Cat#A21206, RRID: AB_2535792 |
| Goat anti-rabbit biotinylated | Dako | Cat#E0432, RRID: AB_2313609 |
| Goat anti-rabbit HRP-conjugated | Dako | Cat#P0448, RRID: AB_2617138 |
| Goat anti-mouse HRP-conjugated | Dako | Cat#P0447, RRID: AB_2617137 |
| Goat anti-rat Alexa 488 | Thermo Fisher | Cat#A11006, RRID: AB_2534074 |
| Goat anti-Rabbit IgG Alexa Fluor 546 | Thermo Fisher | Cat#A-11071, RRID: AB_2534115 |
| Rabbit mAb (DA1E) IgG Isotype Control | Cell Signaling | Cat#011-01, RRID: AB_1550038 |
| Mouse IgG1 | Cell Signaling | Cat#5415S, RRID: AB_10829607 |
| Anti-mouse (Sepharose Bead Conjugate) antibody | Cell Signaling | Cat#3420, RRID: AB_1549744 |
| Anti-rabbit IgG F(ab')2 Fragment (Sepharose Bead Conjugate) antibody | Cell Signaling | Cat#6990, RRID: AB_10828434 |
| APC/Cy7 Rat IgG2b, κ Isotype Ctrl Antibody | Biolegend | Cat#400623, RRID: AB_326565 |
| PE Rat IgG2b, κ Isotype Ctrl Antibody | Biolegend | Cat# 400607, RRID: AB_326551 |
| **Chemicals, Peptides, and Recombinant Proteins** | | |
| Advanced DMEM/F-12 | Gibco/Thermo Fisher | Cat#12634028 |
| B-27™ Supplement (50X), serum free | Gibco/Thermo Fisher | Cat#17504044 |
| CHIR-99021 (CT99021) | Selleckchem | Cat#S1263 |
| Collagenase A | Roche Diagnostics | REF#10103586001 |
| DAPI (4‘.6-diamin-2-phenylindole) | Sigma-Aldrich | Cat#D21490 |
| DMEM (Dulbecco’s Modified Eagle Medium) | Thermo Fisher | Cat#41966052 |
| ECL western blot reagent (250mL) | Thermo Fisher | Cat#32209 |
| Eosin | Roth | Cat#70891 |
| Eukitt mounting medium | Sigma-Aldrich | Cat#03989-100ML |
| Fc-block (human Fcγ-receptors) | BD Biosciences | Cat#564220 |
| Laemmli Sample Buffer (4x) | Bio-Rad | Cat#1610747 |
| Power Block | BioGenex | Cat#HK085-5KE |
| Fluorescence mounting medium | Agilent Technologies | Cat#S302380-2 |
| Glacial acetic acid | Merck | Cat#1.00063.2500 |
| Hematoxylin | Cell Signaling | Cat#MHS80-2.5L |
| IntestiCult™ Organoid Growth Medium (mouse) | STEMCELL Technologies | Cat#06005 |
| Matrigel® Growth Factor Reduced Basement Membrane Matrix | Corning | Cat#354230 |
| N-2 Supplement (100X) | Gibco/Thermo Fisher | Cat# 17502048 |
| OptiPrep density gradient medium | Sigma-Aldrich | Cat#BCBS1952V |
| Recombinant Murine EGF | Peprotech | Cat#315-09 |
| Recombinant Murine Noggin | Peprotech | Cat#250-38 |
| Schiffs reagent | Sigma-Aldrich | Cat#3952016-500ML |
| StemPro Accutase | Thermo Fisher | Cat#A1110501 |
| SYBR green qPCR Kit | Promega | Cat#A6002 |
| Tamoxifen | Sigma-Aldrich | Cat#T5648-1G |
| Trifast peqGOLD | Peqlab | Cat#30-2010 |
| 4-Hydroxytamoxifen (4´OHT) | Sigma-Aldrich | Cat#SML 1666-1ML |
| Penicilin/Streptomycin | Gibco/Thermo Fisher | Cat#15140-122 |
| Blasticidin | Invivogen | Cat#ANT-BL-05 |
| Puromycin | Invivogen | Cat#ANT-PR-1 |
| G418 | Roth | Cat#CP11.1 |
| Valproic acid | Sigma-Aldrich | P4543-10G |
| **Commercial Assays** | | |
| ABC Peroxidase Standard Staining Kit | Thermo Fisher | Cat#32020 |
| DAB-staining Kit | Vector Laboratories | Cat#SK-4100 |
| Envision Detection Systems, Peroxidase/ DAB, Rabbit | Dako | Cat#K4002 |
| Envision Detection Systems, Peroxidase/ DAB, Mouse | Dako | Cat#K4000 |
| GoTaq qPCR Master Mix | Promega | Cat#A600A |
| Qiagen Plasmid Maxi Kit | Qiagen | Cat#12165 |
| RNeasy Mini Kit | Qiagen | Cat#74106 |
| Duolink® In Situ PLA® Probe Anti-Mouse PLUS | Sigma-Aldrich | Cat#DUO92001 |
| Duolink® In Situ PLA® Probe Anti-Rabbit PLUS | Sigma-Aldrich | Cat#DUO92002-30RXN |
| Duolink® In Situ Detection Reagents Green | Sigma-Aldrich | Cat#DUO92014-30RXN |
| Duolink® In Situ Mounting Medium with DAPI | Sigma-Aldrich | Cat#82040-0005 |
| Duolink® In Situ Wash Buffers, Fluorescence | Sigma-Aldrich | Cat#DUO82049 |
| Duolink® In Situ Detection Reagents Green | Sigma-Aldrich | Cat#DUO92014-30RXN |
| Protein G Agarose, Fast Flow | Merck | Cat#16-266 |
| **Experimental Models: Cell Lines** | | |
| Human: HEK293T | ATCC | CRL-3216, RRID: CVCL_0063; |
| Human: HEK 293T R-Spondin | Gift from Dr. Calvin Kuo (Stanford University) | RRID: CVCL_RU08 |
| Human: HeLa | ATCC | CCL-2, RRID: CVCL_0030 |
| Mouse: L-Wnt-3A cells | ATCC | CRL-2647, RRID: CVCL_0635 |
| Mouse: L-Cells | ATCC | CRL-2648, RRID: CVCL_4536 |
| Human: NCI-H1703 | ATCC | CRL-5889, RRID: CVCL_1490 |
| Human: NCI-H1703_*CD44^-/-^* | This paper | N/A |
| Human: HeLa_*CD44^-/-^* | This paper | N/A |
| Human: HEK293T-CD44EGFP | This paper | N/A |
| Human: HEK293T-LRP6mcherry2-CD44EGFP | This paper | N/A |
| Human: HeLa_BC1-TagGFP2 | This paper | N/A |
| Human: HeLa_*CD44^-/-^*_BC1-TagGFP2 | This paper | N/A |
| **Experimental Models: Organisms/Strains** | | |
| Mouse: JAX C57BL/6J Mice | The Jackson Laboratory | MGI:3028467 |
| Mouse: *Villin-CreER^T2^* mice | el Marjou et al., 2004^1^ | MGI:3053826 |
| Mouse: *Cd44* floxed (*Cd44^fl/fl^*) | Shatirishvili et al., 2016^2^ | MGI:6199605 |
| **Oligonucleotides** | | |
| Primers for qPCR, see Table S2 | This paper | N/A |
| Recombinant DNA | | |
| pcDNA3.1 (+) | Invitrogen, Karlsruhe, Germany | Cat#V79020 |
| pCMV-Mesd | Davidson et al., 2005^3^ | N/A |
| pCS2-hLRP6 | Tamai et al., 2000^4^ | N/A |
| pCDNA3.1(+)-hCD44s | Schmitt et al., 2015^5^ | N/A |
| pLV[CRISPR]-hCas9:T2A:Puro-U6>{CD44exon1atg}) | VectorBuilder Inc, CA, USA. | N/A |
| pLV[Exp]-Bsd-EFS>hLRP6 [NM_002336.2](ns):3xGGGGS:{mCherry2}) | VectorBuilder Inc, CA, USA | N/A |
| VB180918-1004rbx (pLV[Exp]-Puro-UBC>hCD44[NM_001001391.1](ns):3xGGGGS:EGFP) | VectorBuilder Inc, CA, USA | N/A |
| M50 Super 8x TOPFlash vector (plasmid 12456) | Addgene | RRID: Addgene_12456 |
| pRL-TK renilla | Promega | RRID: Addgene_27163 |
| Ubi-R-BC1-TagGFP2 | Keller et al., 2018^6^ |  |
| **Software and Algorithms** | | |
| FIJI (Fiji is just ImageJ) | U.S. National Institutes of Health (NIH), Bethesda, MD, US | https://fiji.sc/ (RRID:SCR_002285) |
| Biovoxxel Toolbox (FIJI Plugin) |  | https://www.biovoxxel.de/development/ |
| Zen blue | Zeiss, Jena, Germany | https://www.zeiss.de/mikroskopie/downloads.html |
| FLIMfit 5.1.1 | Photonics Group of the Physics Department, Imperial College London | https://flimfit.org/ |
| FlowJo software (FACS) | BD Biosciences, Heidelberg, Germany | https://www.flowjo.com/  RRID:SCR_008520 |
| StepOne software V2.3 | Life Technologies, Carlsbad, CA, US | https://www.thermofisher.com/de/de/home/technical-resources/software-downloads/StepOne-and-StepOnePlus-Real-Time-PCR-System.html |
| GraphPad Prism 9.0.0 | GraphPad Software, Inc., San Diego, CA, USA | https://www.graphpad.com/scientific-software/prism/  RRID:SCR_002798 |
| Microsoft Excel | Microsoft Corporation, Redmond, WA, USA | RRID:SCR_016137 |
| R | Taiyn and Simko | https///github.com/taiyun/corrplot  RRID: SCR_002394 |
| Keynote | Apple Inc. CA, USA | RRID:SCR_008412 |

***Supplementary Table S2: qPCR Primer***

| Name of Primer | Sequence |
| --- | --- |
| Hsa_β-Actin forward | *5’-TCTGGCACCACACCTTCTACAA-3’* |
| Hsa_β-Actin reverse | *5’-GGCGTACAGGGATAGCACAGC-3’* |
| Mmu_β-Actin forward | 5’-AGGCCAACCGTCAAAAGATGA-3’ |
| Mmu_β-Actin reverse | 5’-GAGGGAGAGCATAGCCCTCGT-3’ |
| Hsa_Axin2 forward | *5’-GGAGAGGGAGAAATGCGTGGA-3’* |
| Hsa_Axin2 reverse | *5’-TAGGTCTTGGTGGCAGGCTTC-3’* |
| Mmu_Axin2 forward | 5´-GTGCAAACTCTCACCCACCGT-3´ |
| Mmu_Axin2 reverse | 5´-CGTCGCTGGATAACTCGCTGT-3´ |
| Hsa_Gapdh forward | *5´-CCCACTCCTCCACCTTTGACG-3´* |
| Hsa_Gapdh reverse | *5´-GTCCACCACCCTGTTGCTGTA-3´* |
| Mmu_Gapdh forward | 5’-ATGTGTCCGTCGTGGATCTGA-3’ |
| Mmu_Gapdh reverse | 5’-TTGCTGTTGAAGTCGCAGGAG-3’ |
| Hsa_NKD forward | *5’-TCTCACCCCGTCTTGCTAAGC-3’* |
| Hsa_NKD reverse | 5’-CTTTGCTCTGCCCACGTCTTG-3’ |
| Mmu_Lyz1 forward | 5’-CCCAGCCTCCAGTCACCATGA-3’ |
| Mmu_Lyz1 reverse | 5’-GCTTGACACCACGGTAGCCAT-3’ |
| Mmu_Defa5 forward | 5’-TGTCCTCCTCTCTGCCCTTGT-3’ |
| Mmu_Defa5 reverse | 5’-CTGGTCCTCTTCCCCTGGCT-3’ |
| Mmu_TNFα forward | 5’-AACTAGTGGTGCCAGCCGATG-3‘ |
| Mmu_TNFα reverse | 5’-ACGGCAGAGAGGAGGTTGACT-3‘ |
| Mmu_Sox9 forward | 5´-ACTCCCAAAACCGACGTGCAA-3´ |
| Mmu_Sox9 reverse | 5´-GTCGCTGCTCAGTTCACCGAT-3´ |
| Mmu_Lgr5 forward | 5´-GCCCTGACCATTGCCACAATC-3´ |
| Mmu_Lgr5 reverse | 5´-GAGCAACACGAGAGCCACCAT-3´ |
| Mmu_Cxcr4 forward | 5´-CCCAGCCCTCCTCCTGACTAT-3´ |
| Mmu_Cxcr4 reverse | 5´-GGGTAAAGGCGGTCACAGATG-3´ |

**References Supplementary Material**

1. el Marjou F, Janssen KP, Chang BH, Li M, Hindie V, Chan L*, et al.* Tissue-specific and inducible Cre-mediated recombination in the gut epithelium. *Genesis* 2004, **39**(3)**:** 186-193.

2. Shatirishvili M, Burk AS, Franz CM, Pace G, Kastilan T, Breuhahn K*, et al.* Epidermal-specific deletion of CD44 reveals a function in keratinocytes in response to mechanical stress. *Cell Death Dis* 2016, **7**(11)**:** e2461.

3. Davidson G, Wu W, Shen J, Bilic J, Fenger U, Stannek P*, et al.* Casein kinase 1 gamma couples Wnt receptor activation to cytoplasmic signal transduction. *Nature* 2005, **438**(7069)**:** 867-872.

4. Tamai K, Semenov M, Kato Y, Spokony R, Liu C, Katsuyama Y*, et al.* LDL-receptor-related proteins in Wnt signal transduction. *Nature* 2000, **407**(6803)**:** 530-535.

5. Schmitt M, Metzger M, Gradl D, Davidson G, Orian-Rousseau V. CD44 functions in Wnt signaling by regulating LRP6 localization and activation. *Cell death and differentiation* 2015, **22**(4)**:** 677-689.

6. Keller BM, Maier J, Secker KA, Egetemaier SM, Parfyonova Y, Rothbauer U*, et al.* Chromobodies to Quantify Changes of Endogenous Protein Concentration in Living Cells. *Mol Cell Proteomics* 2018, **17**(12)**:** 2518-2533.
