# Supplementary figures and images for: Wnt signaling is boosted during intestinal regeneration by a CD44-positive feedback loop
Source: Cell Death Dis. 2022 Feb 21;13(2):168. doi: 10.1038/s41419-022-04607-0 (PMC8861016; doi:10.1038/s41419-022-04607-0)

## Original files Western Blot:

**Figure 6F:** IP Axin, Co-IP CD44

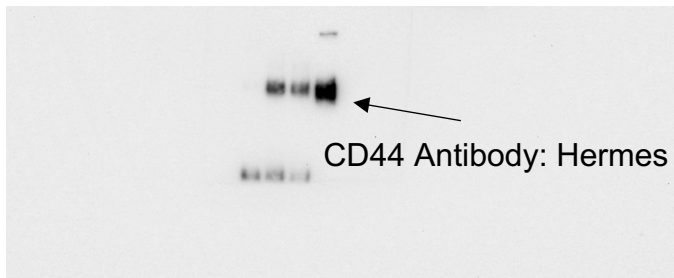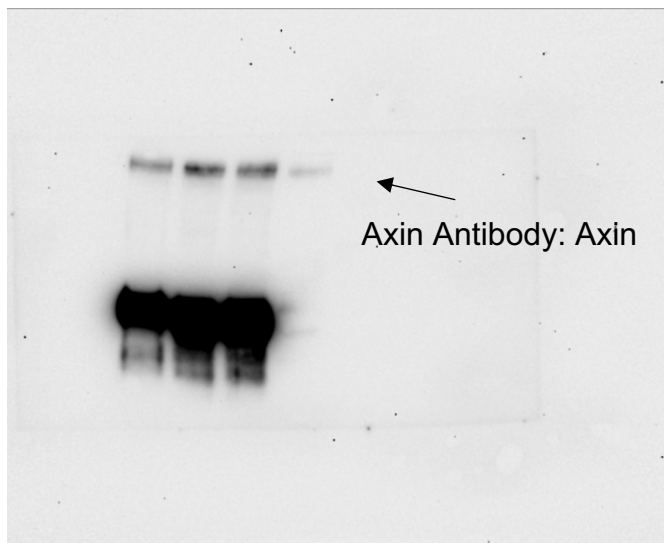

**Figure 6G:** IP CD44, Co-IP DVL

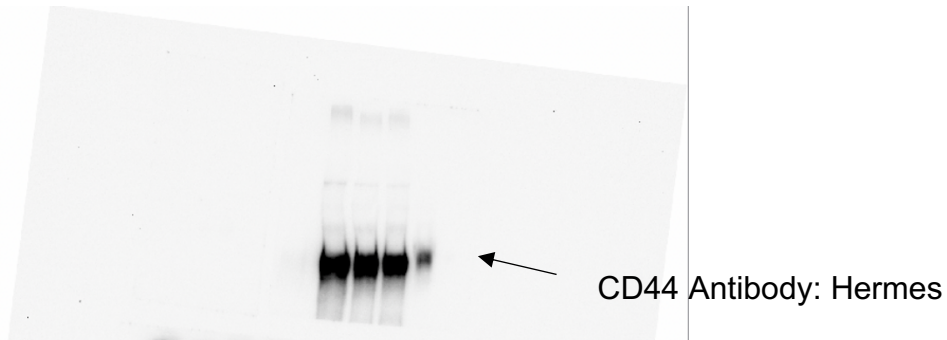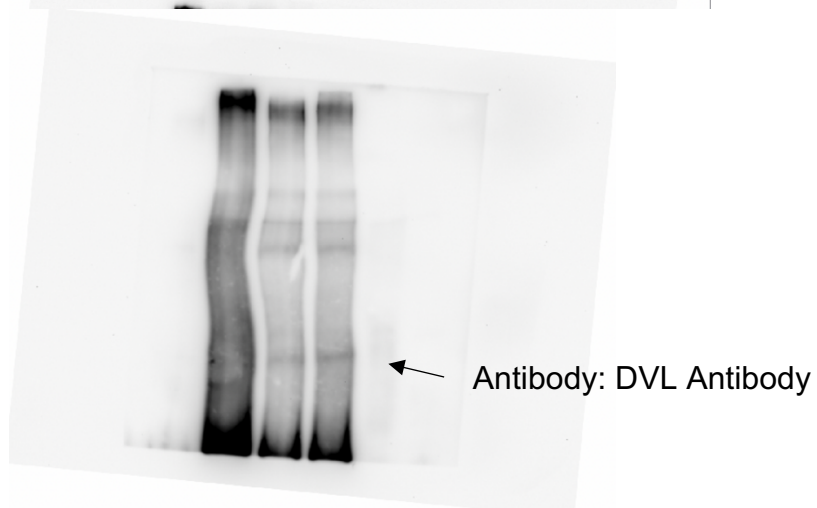

Supplement: Supplementary file 10 — Original Data File [file 41419_2022_4607_MOESM10_ESM.pdf]
