# Supplementary material for: Wnt signaling is boosted during intestinal regeneration by a CD44-positive feedback loop
Source: Cell Death Dis. 2022 Feb 21;13(2):168. doi: 10.1038/s41419-022-04607-0 (PMC8861016; doi:10.1038/s41419-022-04607-0)
Supplement: Supplementary file 13 — declaration of contributions to article [file 41419_2022_4607_MOESM13_ESM.pdf]

DECLARATION OF CONTRIBUTIONS TO ARTICLE

Manuscript Number: CDDIS-21-2626R

AUTHOR FULL NAME	CONTRIBUTION
Romina Judith Walter	designed experiments, conducted most of the experiments and co-wrote the paper
Steffen Joachim Sonnentag	Conceived and performed experiments and analyzed the data
Leonel Munoz-Sagredo	conceived experiments, analyzed the data and co-wrote the paper
Melanie Merkel	performed experiments and analyzed the data
Ludovic Richert	performed experiments and analyzed the data
Felix Bunert	performed experiments and analyzed the data
Yvonne Madelaine Heneka	performed experiments and analyzed the data
Thomas Loustau	performed experiments and analyzed the data
Michael Hodder	performed experiments
Rachel A. Ridgway	performed experiments (supplementary Figure)
Owen J. Sansom	provided expertise, feedback and analyzed the data
Yves Mely	provided expertise, feedback and analyzed the data
Ulrich Rothbauer	contributed with essential reagents and expertise
Mark Schmitt	provided expertise, feedback and analyzed the data
Véronique Orian-Rousseau	conceived the experiments, analyzed the data, secured funding and wrote the manuscript

CONTRIBUTION TO THE FIGURES

FIGURE 1:
1a: RJW (generated the data and prepared the figure), 1b: RJW (generated the data and prepared the figure), 1c: RJW(generated the data and prepared the figure), 1d: MM(generated the data), RJW (prepared the figure), 1e: MH (ISH) and RJW (microscopy, quantification and figure preparation), 1f: RJW (quantification and figure preparation), 1g: RJW (quantification and figure preparation), 1h: RJW (generated the data and prepared the figure).

FIGURE 2:
2a: RJW (generated the data and prepared the figure), 2b: RJW (generated data and prepared the figure), MM (generated data), 2c/d: RJW (passaging results P1,P2 and quantification, figure preparation), SJS (results P0). 2e: RJW (generated the data and prepared the figure), 2f: RJW (generated the data and prepared the figure). 2g: SJS: generated Wnt data and prepared figure), RJW (generated CHIR and prepared figure). LMS (performed statistical analysis).

FIGURE 3:
3a:RJW (prepared the figure) 3b:RJW (generated the data and prepared the figure), MM (generated data). 3c: RJW (generated the data and prepared the figure), 3d:RJW (generated the data and prepared the figure), 3e:RJW (generated the data and prepared the figure), 3f and g: RJW (histological staining and quantification of the HCS, figure preparation). MM (histological staining and quantification of the HCS).

FIGURE 4:
4a: RJW (generated the data and prepared the figure), 4b:RJW (generated the data and prepared the figure), 4c: MM (generated the data) RJW (prepared the figure)

FIGURE 5:
5a: RJW (generated the data and prepared the figure). MM (generated data), 5b: MH (ISH) and RJW (microscopy, quantification and figure preparation), 5c: RJW (analyzed data and prepared the figure). 5d-e: TL (performed analysis and prepared figure).

FIGURE 6:
6a: FB (performed experiment and quantification), RJW: created cell line and prepared the figure. UR: provided plasmid. 6b: RJW (prepared figure), 6c: RJW (performed experiment, quantification and analysis), 6d: RJW (generated DVL data), SJS: (generated AXIN data), 6e: RJW (DVL quantification), SJS: (AXIN quantification). 6f: SJS (performed experiment and prepared figure). 6g:SJS (performed experiment and prepared figure).

FIGURE 7: 7a-c: LR: performed the experiment, microscopic analysis and quantification. RJW created the cell line and performed experiment.

FIGURE 8: RJW drew the graphical abtract.

Date: 17.1.2022 Signature:

Signed for and on behalf of the Author(s): Corresponding author Véronique Orian-Rousseau
