# Supplementary material for: Wnt signaling is boosted during intestinal regeneration by a CD44-positive feedback loop
Source: Cell Death Dis. 2022 Feb 21;13(2):168. doi: 10.1038/s41419-022-04607-0 (PMC8861016; doi:10.1038/s41419-022-04607-0)
Supplement: Supplementary file 12 — Agreement author list [file 41419_2022_4607_MOESM12_ESM.pdf]

From: **Romina Walter** romina3689@web.de
Subject: agreement authorlist
Date: 26. January 2022 at 22:09
To: Orian-Rousseau, Véronique (IBCS) veronique.orian-rousseau@kit.edu

Dear Véronique Orian-Rousseau,

I agree with the addition of Rachel Ridgeway to the list of authors. She contributed to the revision of the paper and performed important experiments.

Best wishes,

Romina Walter

Dr. Romina Walter
Falkenbergerstraße 18
79110 Freiburg
Mobil: 017662306815

From: **Sonnentag, Steffen (IBCS)** steffen.sonnentag@kit.edu
Subject: Re: CDDIS-21-2626RR Publication
Date: 27. January 2022 at 13:02
To: Orian-Rousseau, Véronique (IBCS) veronique.orian-rousseau@kit.edu

Dear Véronique,

I agree to include Rachel Ridgway as an additional author.

Best wishes,

Steffen

Steffen Sonnentag
PhD Student

Karlsruhe Institute of Technology (KIT)
Institute of Biological and Chemical Systems – Functional Molecular Systems (IBCS-FMS)
Hermann-von-Helmholtz-Platz 1, D-76344 Eggenstein-Leopoldshafen
Germany

phone (lab): +49 721 608-23303
phone (office): +49 721 608-24723
steffen.sonnentag@kit.edu

> Am 26.01.2022 um 15:55 schrieb Orian-Rousseau, Véronique (IBCS) <veronique.orian-rousseau@kit.edu>:
>
> Dear all,
>
> As you have noticed, we have added an author to the author list. Rachel Ridgeway performed experiments for the revision.
> CDDis is asking that each of you send a mail to me stating that you agree with this change.
> Can you send me this mail so that we can move on with the publication?
>
> Many thanks
>
> best wishes,
>
> Véronique,

Prof. Dr. Véronique Orian-Rousseau
Group Leader
Karlsruhe Institute of Technology (KIT)

Institute of Biological and Chemical Systems – Functional Molecular Systems (IBCS-FMS)

Campus Nord
Postfach 3640
76021 Karlsruhe
Germany
Tel: +49721-608-26523
Mobile Phone: +491722805799
veronique.orian-rousseau@kit.edu
homepage: https://fms.ibcs.kit.edu/orian-rousseau.php
<first page Walter et al.,.pdf>

From: **Munoz, Leonel (IBCS)** leonel.munoz@partner.kit.edu
Subject: CDDIS-21-2626RR Publication
Date: 26. January 2022 at 16:58
To: Orian-Rousseau, Véronique (IBCS) veronique.orian-rousseau@kit.edu

Dear Professor Orian-Rousseau,

I completely agree to include Rachel Ridgway as a coauthor of our paper since she performed an important experiment for the revision.

Kind regards,
Leonel

Leonel Munoz-Sagredo MD. PhD.
Guest researcher
Research Group: Prof. Dr. Véronique Orian-Rousseau
Institute of Biological and Chemical Systems - Functional Molecular Systems (IBCS-FMS)
Karlsruhe Institute of Technology (KIT)
Hermann-von-Helmholtz-Platz 1,
76344, Eggenstein-Leopoldshafen,
Germany

From: **Merkel, Melanie (ZOO)** melanie.merkel@kit.edu
Subject: Re: CDDIS-21-2626RR Publication
Date: 26. January 2022 at 16:01
To: Orian-Rousseau, Véronique (IBCS) veronique.orian-rousseau@kit.edu

Dear Véronique,

I agree with adding Rachel Ridgway as an author.

Best,
Melanie

> On 26. Jan 2022, at 15:55, Orian-Rousseau, Véronique (IBCS) <veronique.orian-rousseau@kit.edu> wrote:
>
> Dear all,
>
> As you have noticed, we have added an author to the author list. Rachel Ridgeway performed experiments for the revision.
> CDDis is asking that each of you send a mail to me stating that you agree with this change.
> Can you send me this mail so that we can move on with the publication?
>
> Many thanks
>
> best wishes,
>
> Véronique,
>
> Prof. Dr. Véronique Orian-Rousseau
> Group Leader
> Karlsruhe Institute of Technology (KIT)
>
> Institute of Biological and Chemical Systems – Functional Molecular Systems (IBCS-FMS)
>
> Campus Nord
> Postfach 3640
> 76021 Karlsruhe
> Germany
> Tel: +49721-608-26523
> Mobile Phone: +491722805799
> veronique.orian-rousseau@kit.edu
> homepage: https://fms.ibcs.kit.edu/orian-rousseau.php
> <first page Walter et al.,.pdf>

Karlsruher Institute of Technology (KIT)
Department of Cell and Developmental Biology

M. Sc. Melanie Merkel
PhD Student

Fritz-Haber-Weg 4
Building 30.43, Floor 8
76131 Karlsruhe
Germany

Phone: +49 721 608 44195
E-Mail: melanie.merkel@kit.edu
Web: https://zebio.zoo.kit.edu

Registered office:
Kaiserstraße 12, 76131 Karlsruhe

KIT – The Research University in the Helmholtz Association

From: **RICHERT Ludovic (LBP)** ludovic.richert@unistra.fr
Subject: Re: CDDIS-21-2626RR Publication
Date: 26. January 2022 at 16:04
To: veronique orian-rousseau veronique.orian-rousseau@kit.edu

---

Dear Veronique,

I'm agree with the adding of new author (Rachel Ridgeway) on the CDDIS-21-2626RR Publication tittled Wnt signaling is boosted during intestinal regeneration by a CD44-positive feedback loop

Best regards,
Ludovic

Ludovic Richert
Laboratoire de Bioimagerie et Pathologies
CNRS UMR 7021

Bureau E 010 (E 022)
Faculté de pharmacie
74, route du Rhin
67 401 Illkirch-Graffenstaden, France

E-mail: ludovic.richert@unistra.fr
Tel: +33 (0)3 68 85 42 58
Fax: +33 (0)3 68 85 43 13
Website: http://www-lbp.unistra.fr

De: "veronique orian-rousseau" <veronique.orian-rousseau@kit.edu>
À: "Romina Walter" <romina3689@web.de>, "Steffen Sonnentag" <steffen.sonnentag@kit.edu>, "Leo Munoz" <leonel.munoz@partner.kit.edu>, "Merkel, Melanie (ZOO)" <melanie.merkel@kit.edu>, "ludovic richert" <ludovic.richert@unistra.fr>, "Felix Bunert" <felix.bunert97@gmail.com>, "Heneka, Yvonne (IBCS)" <yvonne.heneka@kit.edu>, "Michael Hodder" <m.hodder@beatson.gla.ac.uk>, "Rachel Ridgway" <r.ridgway@beatson.gla.ac.uk>, "Owen Sansom" <o.sansom@beatson.gla.ac.uk>, "yves mely" <yves.mely@unistra.fr>, "Ulrich Rothbauer" <Ulrich.Rothbauer@nmi.de>, "Mark Schmitt" <markschmitt83@gmail.com>
Envoyé: Mercredi 26 Janvier 2022 15:55:51
Objet: CDDIS-21-2626RR Publication

Dear all,

As you have noticed, we have added an author to the author list. Rachel Ridgeway performed experiments for the revision.
CDDis is asking that each of you send a mail to me stating that you agree with this change.
Can you send me this mail so that we can move on with the publication?

Many thanks

best wishes,

Véronique,

Prof. Dr. Véronique Orian-Rousseau
Group Leader
Karlsruhe Institute of Technology (KIT)

Institute of Biological and Chemical Systems – Functional Molecular Systems (IBCS-FMS)

Campus Nord
Postfach 3640
76021 Karlsruhe
Germany
Tel: +49721-608-26523
Mobile Phone: +491722805799
veronique.orian-rousseau@kit.edu
homepage: https://fms.ibcs.kit.edu/orian-rousseau.php

From: **Felix Bunert** felix.bunert97@gmail.com
Subject: Re: CDDIS-21-2626RR Publication
Date: 26. January 2022 at 16:15
To: Véronique veronique.orian-rousseau@kit.edu

---

i agree with this change, mentioned in the email above.

best,

Felix Bunert

Am Mi., 26. Jan. 2022 um 15:56 Uhr schrieb Véronique <veronique.orian-rousseau@kit.edu>:

> Dear all,
>
> As you have noticed, we have added an author to the author list. Rachel Ridgeway performed experiments for the revision.
> CDDis is asking that each of you send a mail to me stating that you agree with this change.
> Can you send me this mail so that we can move on with the publication?
>
> Many thanks
>
> best wishes,
>
> Véronique,
>
> Prof. Dr. Véronique Orian-Rousseau
> Group Leader
> Karlsruhe Institute of Technology (KIT)
>
> Institute of Biological and Chemical Systems – Functional Molecular Systems (IBCS-FMS)
>
> Campus Nord
> Postfach 3640
> 76021 Karlsruhe
> Germany
> Tel: +49721-608-26523
> Mobile Phone: +491722805799
> veronique.orian-rousseau@kit.edu
> homepage: https://fms.ibcs.kit.edu/orian-rousseau.php

From: **Heneka, Yvonne (IBCS)** yvonne.heneka@kit.edu
Subject: AW: CDDIS-21-2626RR Publication
Date: 27. January 2022 at 13:02
To: Orian-Rousseau, Véronique (IBCS) veronique.orian-rousseau@kit.edu

Dear Véronique,

I agree to include Rachel Ridgway as an author to the author list.

Best wishes,

Yvonne

Karlsruher Institut für Technologie (KIT)

Institut für Biologische und Chemische Systeme
Funktionelle Molekulare Systeme (IBCS-FMS)
Yvonne Heneka
PhD Student
Hermann-von-Helmholtz-Platz 1
76344 Eggenstein-Leopoldshafen, Germany

Telefon (Labor): +49 721 608-23303
Telefon (Büro): +49 721 608-24723
E-Mail: yvonne.heneka@kit.edu
Sitz der Körperschaft:
Kaiserstraße 12, 76131 Karlsruhe

KIT – Die Forschungsuniversität in der Helmholtz-Gemeinschaft

---

Von: Orian-Rousseau, Véronique (IBCS)
Gesendet: Mittwoch, 26. Januar 2022 15:55:51
An: Romina Walter; Sonnentag, Steffen (IBCS); Munoz, Leonel (IBCS); Merkel, Melanie (ZOO); RICHERT Ludovic (LBP); Felix Bunert; Heneka, Yvonne (IBCS); Michael Hodder; Rachel Ridgway; Owen Sansom; yves. mely; Ulrich Rothbauer; Mark Schmitt
Betreff: CDDIS-21-2626RR Publication

Dear all,

As you have noticed, we have added an author to the author list. Rachel Ridgeway performed experiments for the revision.
CDDis is asking that each of you send a mail to me stating that you agree with this change.
Can you send me this mail so that we can move on with the publication?

Many thanks

best wishes,

Véronique,

Prof. Dr. Véronique Orian-Rousseau
Group Leader
Karlsruhe Institute of Technology (KIT)

Institute of Biological and Chemical Systems – Functional Molecular Systems (IBCS-FMS)

Campus Nord
Postfach 3640
76021 Karlsruhe
Germany
Tel: +49721-608-26523
Mobile Phone: +491722805799
veronique.orian-rousseau@kit.edu
homepage: https://fms.ibcs.kit.edu/orian-rousseau.php

From: **Loustau, Thomas (IBCS)** thomas.loustau@partner.kit.edu
Subject: RE: CDDIS-21-2626RR Publication: URGENT
Date: 31. January 2022 at 10:57
To: Orian-Rousseau, Véronique (IBCS) veronique.orian-rousseau@kit.edu

---

Dear Veronique,
I agree with the changes and the inclusion of Rachel Ridgway in the list of co-authors of the article CDDIS-21-2626RR.

Best regards

Thomas

De : Orian-Rousseau, Véronique (IBCS)
Envoyé : lundi 31 janvier 2022 10:34:37
À : Loustau, Thomas (IBCS)
Objet : Fwd: CDDIS-21-2626RR Publication: URGENT

Dear Thomas,

Can you answer my email below?

Many thanks,

Véronique,

Prof. Dr. Véronique Orian-Rousseau
Group Leader
Karlsruhe Institute of Technology (KIT)

Institute of Biological and Chemical Systems – Functional Molecular Systems (IBCS-FMS)

Campus Nord
Postfach 3640
76021 Karlsruhe
Germany
Tel: +49721-608-26523
Mobile Phone: +491722805799
veronique.orian-rousseau@kit.edu
homepage: https://fms.ibcs.kit.edu/orian-rousseau.php

> Begin forwarded message:
>
> **From:** Véronique <veronique.orian-rousseau@kit.edu>
> **Subject: CDDIS-21-2626RR Publication**
> **Date:** 26. January 2022 at 15:55:51 CET
> **To:** Romina Walter <romina3689@web.de>, Steffen Sonnentag <steffen.sonnentag@kit.edu>, Leo Munoz <leonel.munoz@partner.kit.edu>, "Merkel, Melanie (ZOO)" <melanie.merkel@kit.edu>, "RICHERT Ludovic (LBP)" <ludovic.richert@unistra.fr>, Felix Bunert <felix.bunert97@gmail.com>, "Heneka, Yvonne (IBCS)" <yvonne.heneka@kit.edu>, Michael Hodder <m.hodder@beatson.gla.ac.uk>, Rachel Ridgway <r.ridgway@beatson.gla.ac.uk>, Owen Sansom <o.sansom@beatson.gla.ac.uk>, "yves. mely" <yves.mely@unistra.fr>, Ulrich Rothbauer <Ulrich.Rothbauer@nmi.de>, Mark Schmitt <markschmitt83@gmail.com>
>
> Dear all,
>
> As you have noticed, we have added an author to the author list. Rachel Ridgeway performed experiments for the revision.
> CDDis is asking that each of you send a mail to me stating that you agree with this change.
> Can you send me this mail so that we can move on with the publication?
>
> Many thanks
>
> best wishes,
>
> Véronique,
>
> Prof. Dr. Véronique Orian-Rousseau

Group Leader
Karlsruhe Institute of Technology (KIT)

Institute of Biological and Chemical Systems – Functional Molecular Systems (IBCS-FMS)

Campus Nord
Postfach 3640
76021 Karlsruhe
Germany
Tel: +49721-608-26523
Mobile Phone: +491722805799
veronique.orian-rousseau@kit.edu
homepage: https://fms.ibcs.kit.edu/orian-rousseau.php

From: **Michael Hodder** hodder1234@hotmail.co.uk
Subject: Re: FW: CDDIS-21-2626RR Publication
Date: 29. January 2022 at 01:19
To: veronique.orian-rousseau@kit.edu
Cc: Owen Sansom o.sansom@beatson.gla.ac.uk, Rachel Ridgway r.ridgway@beatson.gla.ac.uk

Hi all,

I agree with the change

Best Wishes,

Michael

On 28 Jan 2022 12:30, Rachel Ridgway <r.ridgway@beatson.gla.ac.uk> wrote:

> Hi Michael,
>
> Hope you are well.
>
> Please see below about Veronique's publication that you are an author on. Please could you action as soon as possible?
>
> Thanks
>
> Rachel
>
> ---
>
> **From:** Véronique <veronique.orian-rousseau@kit.edu>
> **Sent:** 26 January 2022 14:56
> **To:** Romina Walter <romina3689@web.de>; Steffen Sonnentag <steffen.sonnentag@kit.edu>; Leo Munoz <leonel.munoz@partner.kit.edu>; Merkel, Melanie (ZOO) <melanie.merkel@kit.edu>; RICHERT Ludovic (LBP) <ludovic.richert@unistra.fr>; Felix Bunert <felix.bunert97@gmail.com>; Heneka, Yvonne (IBCS) <yvonne.heneka@kit.edu>; Michael Hodder <m.hodder@beatson.gla.ac.uk>; Rachel Ridgway <r.ridgway@beatson.gla.ac.uk>; Owen Sansom <o.sansom@beatson.gla.ac.uk>; yves. mely <yves.mely@unistra.fr>; Ulrich Rothbauer <Ulrich.Rothbauer@nmi.de>; Mark Schmitt <markschmitt83@gmail.com>
> **Subject:** CDDIS-21-2626RR Publication
>
> Dear all,
>
> As you have noticed, we have added an author to the author list. Rachel Ridgeway performed experiments for the revision.
>
> CDDis is asking that each of you send a mail to me stating that you agree with this change.
>
> Can you send me this mail so that we can move on with the publication?
>
> Many thanks
>
> best wishes,

Véronique,

Prof. Dr. Véronique Orian-Rousseau
Group Leader
Karlsruhe Institute of Technology (KIT)

Institute of Biological and Chemical Systems – Functional Molecular Systems (IBCS-FMS)

Campus Nord
Postfach 3640
76021 Karlsruhe
Germany
Tel: +49721-608-26523
Mobile Phone: +491722805799
veronique.orian-rousseau@kit.edu
homepage: https://fms.ibcs.kit.edu/orian-rousseau.php

From: **Owen Sansom** o.sansom@beatson.gla.ac.uk
Subject: RE: CDDIS-21-2626RR Publication
Date: 26. January 2022 at 17:02
To: Sonnentag, Steffen (IBCS) veronique.orian-rousseau@kit.edu

Hi Veronique
Im happy to confirm that Rachel Ridgway should be added to the paper..

Cheers
Owen

From: Véronique <veronique.orian-rousseau@kit.edu>
Sent: 26 January 2022 14:56
To: Romina Walter <romina3689@web.de>; Steffen Sonnentag <steffen.sonnentag@kit.edu>; Leo Munoz <leonel.munoz@partner.kit.edu>; Merkel, Melanie (ZOO) <melanie.merkel@kit.edu>; RICHERT Ludovic (LBP) <ludovic.richert@unistra.fr>; Felix Bunert <felix.bunert97@gmail.com>; Heneka, Yvonne (IBCS) <yvonne.heneka@kit.edu>; Michael Hodder <m.hodder@beatson.gla.ac.uk>; Rachel Ridgway <r.ridgway@beatson.gla.ac.uk>; Owen Sansom <o.sansom@beatson.gla.ac.uk>; yves. mely <yves.mely@unistra.fr>; Ulrich Rothbauer <Ulrich.Rothbauer@nmi.de>; Mark Schmitt <markschmitt83@gmail.com>
Subject: CDDIS-21-2626RR Publication

Dear all,

As you have noticed, we have added an author to the author list. Rachel Ridgeway performed experiments for the revision.
CDDis is asking that each of you send a mail to me stating that you agree with this change.
Can you send me this mail so that we can move on with the publication?

Many thanks

best wishes,

Véronique,

Prof. Dr. Véronique Orian-Rousseau
Group Leader
Karlsruhe Institute of Technology (KIT)

Institute of Biological and Chemical Systems – Functional Molecular Systems (IBCS-FMS)

Campus Nord
Postfach 3640
76021 Karlsruhe
Germany
Tel: +49721-608-26523
Mobile Phone: +491722805799
veronique.orian-rousseau@kit.edu
homepage: https://fms.ibcs.kit.edu/orian-rousseau.php

From: **MELY Yves (PHA)** yves.mely@unistra.fr
Subject: RE: CDDIS-21-2626RR Publication
Date: 26. January 2022 at 22:55
To: Véronique veronique.orian-rousseau@kit.edu

Dear Veronique,
I agree that Rachel Ridgeway is added as co-author on the paper.
Best regards
Yves

De : Véronique [mailto:veronique.orian-rousseau@kit.edu]
Envoyé : mercredi 26 janvier 2022 15:56
À : Romina Walter <romina3689@web.de>; Steffen Sonnentag <steffen.sonnentag@kit.edu>; Leo Munoz <leonel.munoz@partner.kit.edu>; Merkel, Melanie (ZOO) <melanie.merkel@kit.edu>; RICHERT Ludovic (LBP) <ludovic.richert@unistra.fr>; Felix Bunert <felix.bunert97@gmail.com>; Heneka, Yvonne (IBCS) <yvonne.heneka@kit.edu>; Michael Hodder <m.hodder@beatson.gla.ac.uk>; Rachel Ridgway <r.ridgway@beatson.gla.ac.uk>; Owen Sansom <o.sansom@beatson.gla.ac.uk>; yves. mely <yves.mely@unistra.fr>; Ulrich Rothbauer <Ulrich.Rothbauer@nmi.de>; Mark Schmitt <markschmitt83@gmail.com>
Objet : CDDIS-21-2626RR Publication

Dear all,

As you have noticed, we have added an author to the author list. Rachel Ridgeway performed experiments for the revision.
CDDis is asking that each of you send a mail to me stating that you agree with this change.
Can you send me this mail so that we can move on with the publication?

Many thanks

best wishes,

Véronique,

Prof. Dr. Véronique Orian-Rousseau
Group Leader
Karlsruhe Institute of Technology (KIT)

Institute of Biological and Chemical Systems – Functional Molecular Systems (IBCS-FMS)

Campus Nord
Postfach 3640
76021 Karlsruhe
Germany
Tel: +49721-608-26523
Mobile Phone: +491722805799
veronique.orian-rousseau@kit.edu

homepage: https://fms.ibcs.kit.edu/orian-rousseau.php

From: **Rothbauer, Ulrich** Ulrich.Rothbauer@nmi.de
Subject: AW: CDDIS-21-2626RR Publication
Date: 26. January 2022 at 16:10
To: Véronique veronique.orian-rousseau@kit.edu

Dear Veronique,
Within I agree that Rachel Ridgeway is added to the list of authors
Kind regards
Ulrich Rothbauer

Von: Véronique [mailto:veronique.orian-rousseau@kit.edu]
Gesendet: Mittwoch, 26. Januar 2022 15:56
An: Romina Walter <romina3689@web.de>; Steffen Sonnentag <steffen.sonnentag@kit.edu>; Leo Munoz <leonel.munoz@partner.kit.edu>; Merkel, Melanie (ZOO) <melanie.merkel@kit.edu>; RICHERT Ludovic (LBP) <ludovic.richert@unistra.fr>; Felix Bunert <felix.bunert97@gmail.com>; Heneka, Yvonne (IBCS) <yvonne.heneka@kit.edu>; Michael Hodder <m.hodder@beatson.gla.ac.uk>; Rachel Ridgway <r.ridgway@beatson.gla.ac.uk>; Owen Sansom <o.sansom@beatson.gla.ac.uk>; yves. mely <yves.mely@unistra.fr>; Rothbauer, Ulrich <Ulrich.Rothbauer@nmi.de>; Mark Schmitt <markschmitt83@gmail.com>
Betreff: CDDIS-21-2626RR Publication

Dear all,

As you have noticed, we have added an author to the author list. Rachel Ridgeway performed experiments for the revision.
CDDis is asking that each of you send a mail to me stating that you agree with this change.
Can you send me this mail so that we can move on with the publication?

Many thanks

best wishes,

Véronique,

Prof. Dr. Véronique Orian-Rousseau
Group Leader
Karlsruhe Institute of Technology (KIT)

Institute of Biological and Chemical Systems – Functional Molecular Systems (IBCS-FMS)

Campus Nord
Postfach 3640
76021 Karlsruhe
Germany
Tel: +49721-608-26523
Mobile Phone: +491722805799
veronique.orian-rousseau@kit.edu
homepage: https://fms.ibcs.kit.edu/orian-rousseau.php

From: **Mark Schmitt** markschmitt83@gmail.com
Subject: Re: CDDIS-21-2626RR Publication
Date: 26. January 2022 at 17:43
To: Véronique veronique.orian-rousseau@kit.edu

Hi Véro,

I agree with including Rachel Ridgway in the list of authors of the manuscript.

Best

Mark

Véronique <veronique.orian-rousseau@kit.edu> schrieb am Mi., 26. Jan. 2022, 15:56:

> Dear all,
>
> As you have noticed, we have added an author to the author list. Rachel Ridgeway performed experiments for the revision.
> CDDis is asking that each of you send a mail to me stating that you agree with this change.
> Can you send me this mail so that we can move on with the publication?
>
> Many thanks
>
> best wishes,
>
> Véronique,
>
> Prof. Dr. Véronique Orian-Rousseau
> Group Leader
> Karlsruhe Institute of Technology (KIT)
>
> Institute of Biological and Chemical Systems – Functional Molecular Systems (IBCS-FMS)
>
> Campus Nord
> Postfach 3640
> 76021 Karlsruhe
> Germany
> Tel: +49721-608-26523
> Mobile Phone: +491722805799
> veronique.orian-rousseau@kit.edu
> homepage: https://fms.ibcs.kit.edu/orian-rousseau.php
