# Supplementary material for: Wnt signaling is boosted during intestinal regeneration by a CD44-positive feedback loop
Source: Cell Death Dis. 2022 Feb 21;13(2):168. doi: 10.1038/s41419-022-04607-0 (PMC8861016; doi:10.1038/s41419-022-04607-0)
Supplement: Supplementary file 14 — Cover letter with competing interest statement [file 41419_2022_4607_MOESM14_ESM.pdf]

Karlsruher Institut für Technologie

Institute of Biological and Chemical Systems
– Functional Molecular Systems (IBCS-FMS)

Prof. Dr Véronique Orian-Rousseau

Hermann-von-Helmholtz Platz 1
76344 Eggenstein-Leopoldshafen, Germany
Telephone: +49 721 608-26523
Fax: +49 721 608-23354

E-Mail: veronique.orian-rousseau@kit.edu
Date: 30.12.2021

KIT-Campus North | PO 6980 | 76344 Eggenstein-Leopoldshafen

Zertifikat seit 2010
audit familiengerechte hochschule

Dear Professor Aberdam,

We are very thankful to you for having given us the opportunity to resubmit the paper entitled “Wnt signaling is boosted during intestinal regeneration by a CD44-positive feedback loop” by Walter et al. We thank the reviewers for the detailed analysis of our paper and the suggested experiments. We are grateful for the constructive ideas and the kind tone of the comments.
We have addressed most of the points raised by the reviewers and several of the added experiments suggested by the reviewers strongly support our hypothesis of a direct function of CD44 at the Wnt signalosome of intestinal stem cells. Therefore, we hope that our paper is now suitable for publication in Cell death and Disease.
One additional author, Rachel Ridgeway was added to the author list since she performed an additional experiment.

Best regards,

Véronique Orian-Rousseau

PS: V. OR is an advisory board member and has shares in amcure. amcure develops a CD44v6 peptide. CD44v6 is not the subject of this study and the peptide was not used in this paper.
